# Calcineurin Plays Key Roles in the Dimorphic Transition and Virulence of the Human Pathogenic Zygomycete *Mucor circinelloides*

**DOI:** 10.1371/journal.ppat.1003625

**Published:** 2013-09-05

**Authors:** Soo Chan Lee, Alicia Li, Silvia Calo, Joseph Heitman

**Affiliations:** Department of Molecular Genetics and Microbiology, Duke University Medical Center, Durham, North Carolina, United States of America; University of Melbourne, Australia

## Abstract

Many pathogenic fungi are dimorphic and switch between yeast and filamentous states. This switch alters host-microbe interactions and is critical for pathogenicity. However, in zygomycetes, whether dimorphism contributes to virulence is a central unanswered question. The pathogenic zygomycete *Mucor circinelloides* exhibits hyphal growth in aerobic conditions but switches to multi-budded yeast growth under anaerobic/high CO_2_ conditions. We found that in the presence of the calcineurin inhibitor FK506, *Mucor* exhibits exclusively multi-budded yeast growth. We also found that *M. circinelloides* encodes three calcineurin catalytic A subunits (CnaA, CnaB, and CnaC) and one calcineurin regulatory B subunit (CnbR). Mutations in the latch region of CnbR and in the FKBP12-FK506 binding domain of CnaA result in hyphal growth of *Mucor* in the presence of FK506. Disruption of the *cnbR* gene encoding the sole calcineurin B subunit necessary for calcineurin activity yielded mutants locked in permanent yeast phase growth. These findings reveal that the calcineurin pathway plays key roles in the dimorphic transition from yeast to hyphae. The *cnbR* yeast-locked mutants are less virulent than the wild-type strain in a heterologous host system, providing evidence that hyphae or the yeast-hyphal transition are linked to virulence. Protein kinase A activity (PKA) is elevated during yeast growth under anaerobic conditions, in the presence of FK506, or in the yeast-locked *cnbR* mutants, suggesting a novel connection between PKA and calcineurin. *cnaA* mutants lacking the CnaA catalytic subunit are hypersensitive to calcineurin inhibitors, display a hyphal polarity defect, and produce a mixture of yeast and hyphae in aerobic culture. The *cnaA* mutants also produce spores that are larger than wild-type, and spore size is correlated with virulence potential. Our results demonstrate that the calcineurin pathway orchestrates the yeast-hyphal and spore size dimorphic transitions that contribute to virulence of this common zygomycete fungal pathogen.

## Author Summary

Calcineurin is a Ca^2+^/calmodulin-dependent, serine/threonine-specific protein phosphatase. In pathogenic fungi, calcineurin is involved in morphogenesis and virulence. Therefore, calcineurin is an attractive antifungal drug target. The roles of calcineurin in virulence have been established in both major human pathogenic fungi (*Candida* species, *Cryptococcus neoformans/gattii*, *Aspergillus fumigatus*) and in plant pathogenic fungi (*Magnaporthe oryzae*, *Ustilago maydis/hordei*). However, the role of calcineurin is currently unknown in pathogenic zygomycetes. We found that the calcineurin inhibitors FK506 and cyclosporine A inhibit the growth of a prevalent zygomycete pathogen, *Mucor*. This fungus grows as multi-budded yeast under anaerobic conditions, and we have found that even in aerated culture (which without FK506 would result in abundant hyphal growth), *Mucor* exhibits yeast growth when exposed to FK506. *Mucor cnbR* mutants that lack the calcineurin regulatory subunit essential for calcineurin activity, are locked in perpetual yeast phase growth, indicating that calcineurin is required for hyphal growth. We further demonstrated that these yeast-locked mutants are attenuated for virulence, illustrating that hyphae or the yeast-hyphal transition are linked to virulence. These findings indicate that: 1) calcineurin governs the yeast/hyphae morphogenic transition; 2) a link exists between respiration and the calcineurin pathway; and 3) calcineurin inhibitors are attractive anti-mucormycosis drug candidates.

## Introduction

*Mucor circinelloides* is one causal agent of mucormycosis, an uncommon but frequently lethal fungal infection of humans. Several other species belonging to the Mucorales order also cause mucormycosis, including some *Rhizopus* species, *Absidia*, *Cunninghamella*, *Rhizomucor*, *Apophysomyces trapeziformis*, and others [1]–[3]. Mucormycosis is an emerging fungal infection often afflicting immunocompromised and vulnerable populations including patients with diabetes, AIDS, hematologic malignancies, or trauma [3]–[8]. Susceptible hosts also include patients with solid organ transplants and those with high serum iron levels [4], [8], [9]. Mucormycosis is the second most common fungal infection among patients with hematological malignancies and transplants. This infection is associated with high mortality rates [4], [8], [10]: ∼50% for all mucormycosis infections and >90% for disseminated infections [4]–[6], [9], [11]. Recently, a cluster of mucormycosis cases was reported among the tornado victims in Joplin, MO in the spring of 2011; 13 cases were reported with five fatalities. Based on 28S ribosomal DNA sequence the responsible species has been identified as *A. trapeziformis* [3]. Despite the increasing incidence of disease, high mortality rate, and unmet clinical needs for therapy, zygomycetes are understudied compared to other pathogenic fungi.

*Mucor* species are dimorphic fungi and exhibit either hyphal or yeast growth depending upon the conditions (reviewed in [12]). Hyphal growth of *Mucor* was first thought to originate from species of *Saccharomyces* by transmutation [13] until Louis Pasteur discovered that *Mucor* grows as a multi-budded yeast under anaerobic/high CO_2_ growth conditions [14]. Later, Bartnicki-Garcia and Nickerson rediscovered the induction of *Mucor* yeast growth by CO_2_ [15]–[17]. Although dimorphic *Mucor* species vary in their responses to the environment during morphogenic changes, common critical factors that induce yeast growth of *Mucor* species include oxygen concentration, CO_2_ concentration, and carbon source (reviewed in [12]). In addition, several chemicals that inhibit mitochondrial function [including potassium cyanide and antimycin A (which block electron transport) or oligomycin and phenyl alcohol (which inhibit oxidative phosphorylation)] induce yeast growth in *Mucor* spp., even in aerobic conditions [18]–[20]. Inhibition of the synthesis of cytochrome b and other mitochondrial components by chloramphenicol also results in *Mucor* yeast growth [21], [22]. Thus, respiration, anaerobic conditions, and high CO_2_ environments all contribute to the yeast-hyphal transition. Cerulenin, a lipid metabolism inhibitor, and cycloleucine, an S-adenosylmethionine (SAM) synthetase inhibitor, both block the yeast to hyphal growth transition under aerobic conditions [23], [24]. Notably, adding cyclic AMP (cAMP) to the culture medium induces yeast growth of *Mucor* spp. (reviewed in [12] and references therein). cAMP activates cAMP-dependent protein kinase A (PKA), implicating a role for protein kinase A in the *Mucor* dimorphic transition, and this is supported by a series of recent studies, including genetic analyses in which genes encoding PKA regulatory subunits were disrupted by homologous recombination [25]–[31]. PKA also plays a key role in various morphogenesis processes including germination, branching, and polarized growth in *Mucor* [26], [32]–[37]

Dimorphism has evolved in multiple lineages in the fungal kingdom, including ascomycetes, basidiomycetes, and zygomycetes [38]–[40]. In many pathogenic fungi, the morphogenic transition is closely related to pathogenicity. A group of so-called “dimorphic fungi” grow as molds at lower environmental temperatures; however, in association with the host these fungi undergo a thermal dimorphic switch, resulting in yeast growth as virulent forms at 37°C (reviewed in [41], [42]). The dimorphic fungal pathogens include *Histoplasma capsulatum*, *Paracoccidioides brasiliensis*, *Coccidioides immitis*/*posadasii*, *Blastomyces dermatitidis*, and *Penicillium marneffei*, among others. The most prevalent fungal commensal and pathogen, *Candida albicans*, exhibits yeast and hyphal growth, and the ability of this fungus to grow as both forms is necessary for virulence; yeast-locked or hyphal-locked mutants are both avirulent [39], [40], [43], [44]. The basidiomycete human pathogen *Cryptococcus neoformans* changes its morphology; it grows mainly as yeast but during the sexual cycle the fungus forms hyphae and produces sexual spores that are readily disseminated as infectious propagules [45]–[47]. *C. neoformans* also exhibits pseudohyphal growth, which is advantageous for escape from amoebae, one of its natural predators [48], [49]. In the plant pathogenic basidiomycete *Ustilago maydis*, the yeast form is not virulent; however, the hyphae produced by mating cause disease on plant hosts and transform infected plant tissue into galls [50]. Therefore, morphogenic transitions play major roles during host infection by pathogenic fungi. Dimorphism in zygomycetes, especially in *Mucor* spp. was studied decades ago; however, our knowledge of its contributions to pathogenesis is limited and the underlying genetics, beyond the involvement of anaerobic/high CO_2_, respiration, and PKA is not well established.

Our study reveals that calcineurin plays a crucial role during the yeast-hyphal growth transition of *M. circinelloides*. The calcineurin pathway is conserved throughout eukaryotes. Calcineurin is a Ca^2+^/calmodulin-dependent serine/threonine specific protein phosphatase that consists of two subunits: the catalytic A subunit, which has phosphatase activity, and the regulatory B subunit, which binds calcium and the A subunit to activate the enzyme complex. Both subunits are required for enzyme activity. The drugs tacrolimus (FK506) and cyclosporine A (CsA) form complexes with the immunophilins FKBP12 and cyclophilin A, respectively. The protein-drug complexes bind to the hydrophobic interface between the calcineurin A and B subunits to inhibit phosphatase activity [51], [52]. We found that FK506 inhibits hyphal growth and instead induces yeast growth in the pathogenic zygomycete *Mucor*. Gain of function mutations in the calcineurin regulatory B and catalytic A subunit genes were identified that confer resistance to FK506. Furthermore, disruption of the calcineurin regulatory B subunit required for calcineurin activity results in a yeast-locked phenotype, supporting our conclusion that calcineurin regulates the dimorphic transition. Notably, the *cnbRΔ* yeast phase locked mutants were less virulent, suggesting that either hyphae are more virulent than yeast or the morphogenic switch is central to pathogenicity of this fungus. In addition, cAMP-dependent protein kinase A activity was found to be elevated during yeast compared to hyphal growth, and also when calcineurin activity was inhibited by FK506 or mutation. We also show that calcineurin is involved in hyphal polarity and spore size dimorphism, which is linked to virulence [53].

Calcineurin is involved in the morphogenesis and virulence of multiple pathogenic fungi (reviewed in [54], [55]): in *C. neoformans*, calcineurin is required for growth at 37°C [56]; in *Candida* spp., calcineurin functions in antifungal drug resistance/tolerance, survival in serum, and virulence [57]–[60]; calcineurin plays a role in morphogenesis in *P. brasiliensis* [61]; and in *Aspergillus fumigatus*, calcineurin regulates morphogenesis and thereby pathogenesis [62]. Therefore, calcineurin is a promising antifungal drug target and this study provides a novel foundation to develop approaches to control the emerging fungal infection mucormycosis.

## Results

### The calcineurin inhibitor FK506 induces *M. circinelloides* to grow as yeast

**Figure 1:**
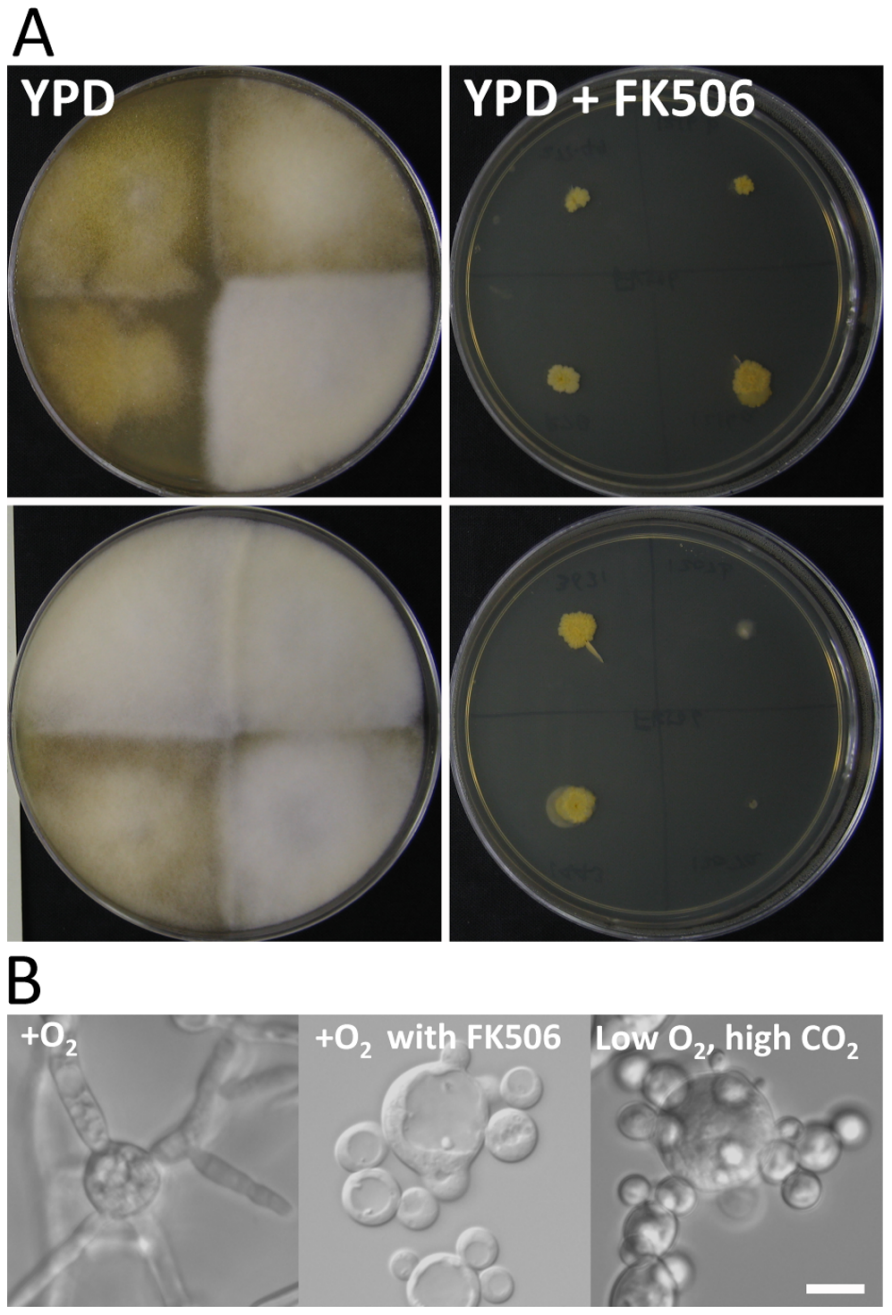
The calcineurin inhibitor FK506 blocks hyphal growth and enforces multi-budded yeast growth in *M.* *circinelloides*. (A) On YPD agar media, eight *M. circinelloides* isolates (upper: CBS277.49, ATCC1216b, R7B, and ATCC1216a, bottom: NRRL3631, ATCC1207b, NRRL1443, ATCC1207a) grow as hyphae, whereas in the presence of FK506 (1 µg/mL), they form a compact yeast colony. (B) In liquid YPD media with vigorous shaking for aeration, wild-type exhibits hyphal growth. When FK506 is present in the media, *Mucor* grows only as a multi-budded yeast, as is also observed during growth under low oxygen/high CO_2_ conditions (isolate grown at the bottom of a flask completely filled with liquid YPD media generating microaerobic/high CO_2_ conditions). Representative images with isolate CBS277.49 are shown here. Similar results were observed with other *Mucor* isolates (data not shown). Scale = 5 µm.

We found that in *Mucor* the calcineurin inhibitor FK506 inhibits hyphal growth and drives multi-budded yeast growth (Figure 1 and Videos S1 and S2). Eight different *M. circinelloides* isolates (10^3^ spores) were each inoculated aerobically on YPD agar media or YPD agar media containing FK506 (1 µg/mL). At 4 days post-inoculation, the colonies grown on YPD with FK506 were significantly smaller than those on YPD alone (Figure 1A). The smaller, compact colonies consisted entirely of yeast form *Mucor* (data not shown). In liquid culture, with vigorous shaking for aeration, the *Mucor* isolate CBS277.49 grew exclusively as a mold (Figure 1B); however, this isolate exhibited only yeast growth when cultured in the same fashion but in the presence of FK506 (1 µg/mL). The multi-budded yeast phenotype imposed by FK506 phenocopies *Mucor* yeast growth under anaerobic/high CO_2_ growth conditions (Figure 1B). This observation indicates that there is a link between respiration/CO_2_ sensing and the calcineurin pathway. On the other hand, another calcineurin inhibitor cyclosporine A (CsA) did not induce multi-budded yeast growth, and instead resulted in abnormal, stunted hyphal growth (Figure S1). Higher concentrations of CsA did not produce yeast growth (data not shown). This result suggests that FK506-specific calcineurin inhibition plays a role in the hyphal-yeast switch. We note that although CsA does stunt hyphal growth, the efficacy may not be sufficient to enforce yeast growth compared to FK506 if levels of the cyclophilin A-CsA complex are insufficient to inhibit all calcineurin activity. This hypothesis is supported by findings that 1) in a sensitized background in which calcineurin activity has been reduced with a sub-inhibitory concentration of FK506, CsA is now fully able to block the dimorphic transition and impose yeast growth (Figures S2 and 2) in regulatory B subunit mutant *CNBR-1* (N125Y, note that the all gain-of-function mutations are designated with capital letters) mutants isolated as being resistant to FK506 (described below), CsA imposes a multi-budded yeast phenotype (See supplemental figure S5).

**Figure 2:**
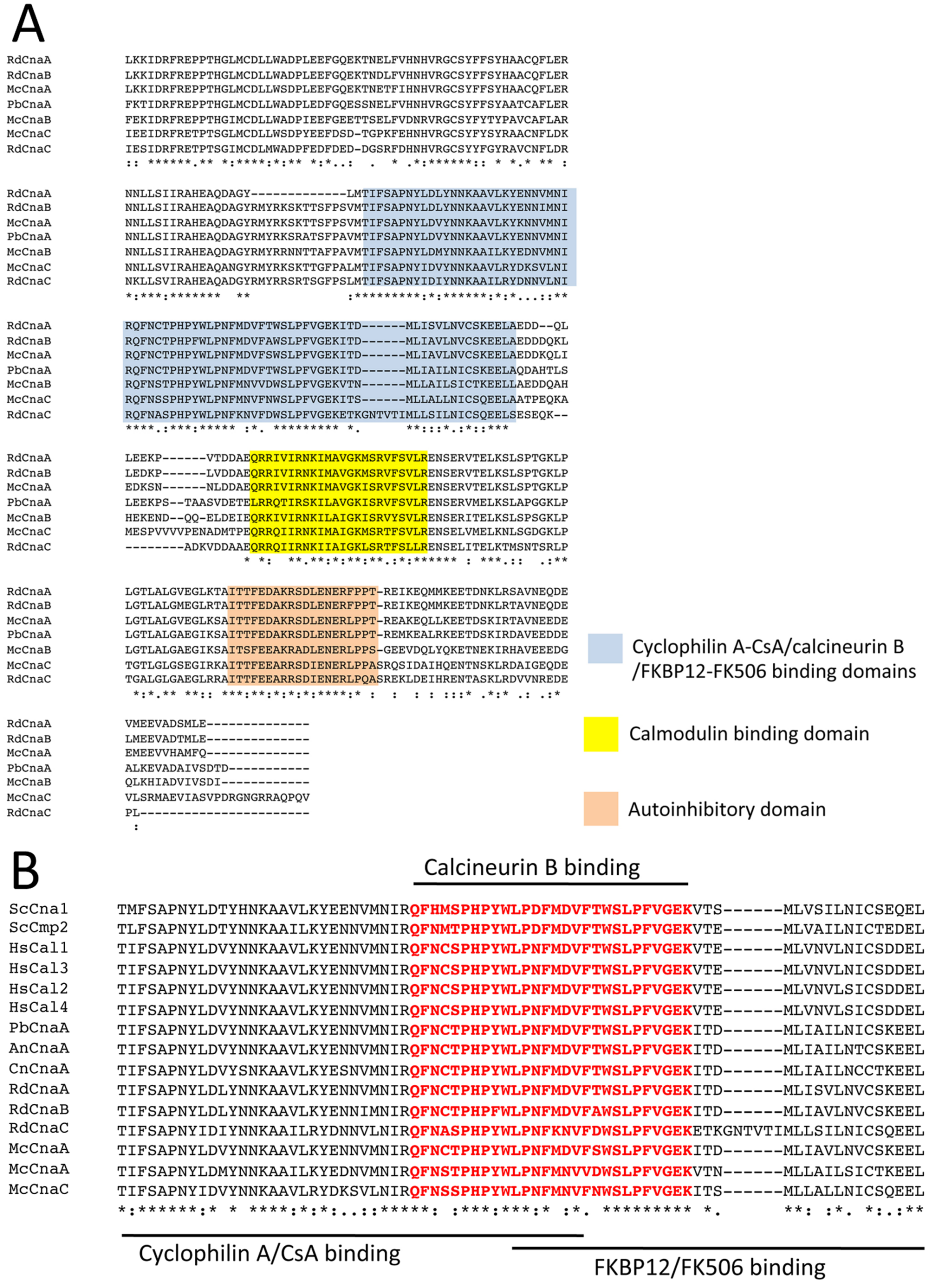
Conserved domains of the three catalytic A subunits of *M.* *circinelloides*. (A) The calcineurin catalytic A subunits of *M. circinelloides*, *R. delemar*, and *P. blakesleeanus* have a highly conserved N-terminal phosphatase catalytic domain followed by a unique C-terminal region with domains mediating binding to 1) the calcineurin B regulatory subunit; 2) cyclophilin A-CsA complex; 3) FKBP12-FK506 complex (depicted as blue box and in B panel); and 4) calmodulin (yellow box), and an auto-inhibitory domain (orange box) that docks into the active site. This C-terminal regulatory domain distinguishes calcineurin from all other known protein phosphatases. (B) The calcineurin B subunit binding domain is in red font. Asterisk is for identical amino acids, colon is for conservative amino acid substitutions, and period is for semiconservative amino acid substitutions. Hs: *Homo sapiens*, Sc: *S. cerevisiae*, Cn: *C. neoformans*, An: *A. nidulans*, Mc: *M. circinelloides*, Rd: *R. delemar*, and Pb: *Phycomyces blakesleeanus*.

### Identification of calcineurin pathway components in the *Mucor* genome

We identified calcineurin pathway components in the *M. circinelloides* genome. Unusually high numbers of calmodulin and calmodulin (CAM) kinase orthologs (nine calmodulins) were identified in the genomes with six CAM kinases Figure S3). Interestingly, the *Mucor* genome encodes three calcineurin catalytic A subunits along with a single calcineurin regulatory B subunit, FKBP12, and cyclophilin A.

We further examined the three catalytic A subunit (*cna*) genes and found that the Cna ortholog proteins contain an N-terminal phosphatase domain and a C-terminal regulatory domain containing the calcineurin B subunit binding domain, cyclophilin A-cyclosporine A complex binding domain, FKBP12-FK506 complex binding domain (the domains were determined based on [63]), calmodulin binding domain, and auto-inhibitory domain (Figure 2), further demonstrating that the identified Cna proteins are *bona fide* calcineurin catalytic A subunits. The sequences of the Cna proteins are distinct, and there is 73% identity shared between CnaA and CnaB; 64% identity between CnaA and CnaC; and 62% identity between CnaB and CnaC. It is interesting to consider how multiple paralogs of the *cna* genes evolved in the zygomycetes (Figure S3). In phylogenetic analyses, the three zygomycete species including *M. circinelloides*, *Rhizopus delemar*, and *Phycomyces blakesleeanus*, are conserved on a common branch, including McCnaA, PbCnaA, RdCnaA, and RdCnaB. This observation suggests that the calcineurin A subunit gene in this group may be the more ancestral (Figure S3).

### Gain-of-function mutations in the *cnaA* and *cnbR* genes result in hyphal growth in the presence of FK506

FKBP12 is an immunophilin family protein with *cis-trans* peptide prolyl isomerase activity that serves as the cellular receptor for FK506 and rapamycin [64]. When bound to FK506, FKBP12 binds to the interface between the calcineurin catalytic A and regulatory B subunits, inhibiting phosphatase activity by occluding substrate access to the active site [51]. FKBP12 also binds to rapamycin to inhibit the Tor pathway. Disruption of the gene encoding FKBP12 confers resistance to FK506 and rapamycin (in the absence of FKBP12, FK506 fails to bind calcineurin) [65]. Amino acid substitutions in the calcineurin regulatory B and catalytic A subunit surface that interact with the FKBP12-FK506 complex result in resistance to FK506 [63], [66]. Calcineurin FK506 resistant mutants expressing FKBP12 remain rapamycin sensitive. Sensitivity is defined as FK506 enforced yeast growth or rapamycin limiting hyphal growth of the fungus; resistance to FK506 is defined as strains forming hyphae instead of yeast in the presence of FK506, and resistance to rapamycin as strains growing as in non-drug media.

**Figure 3:**
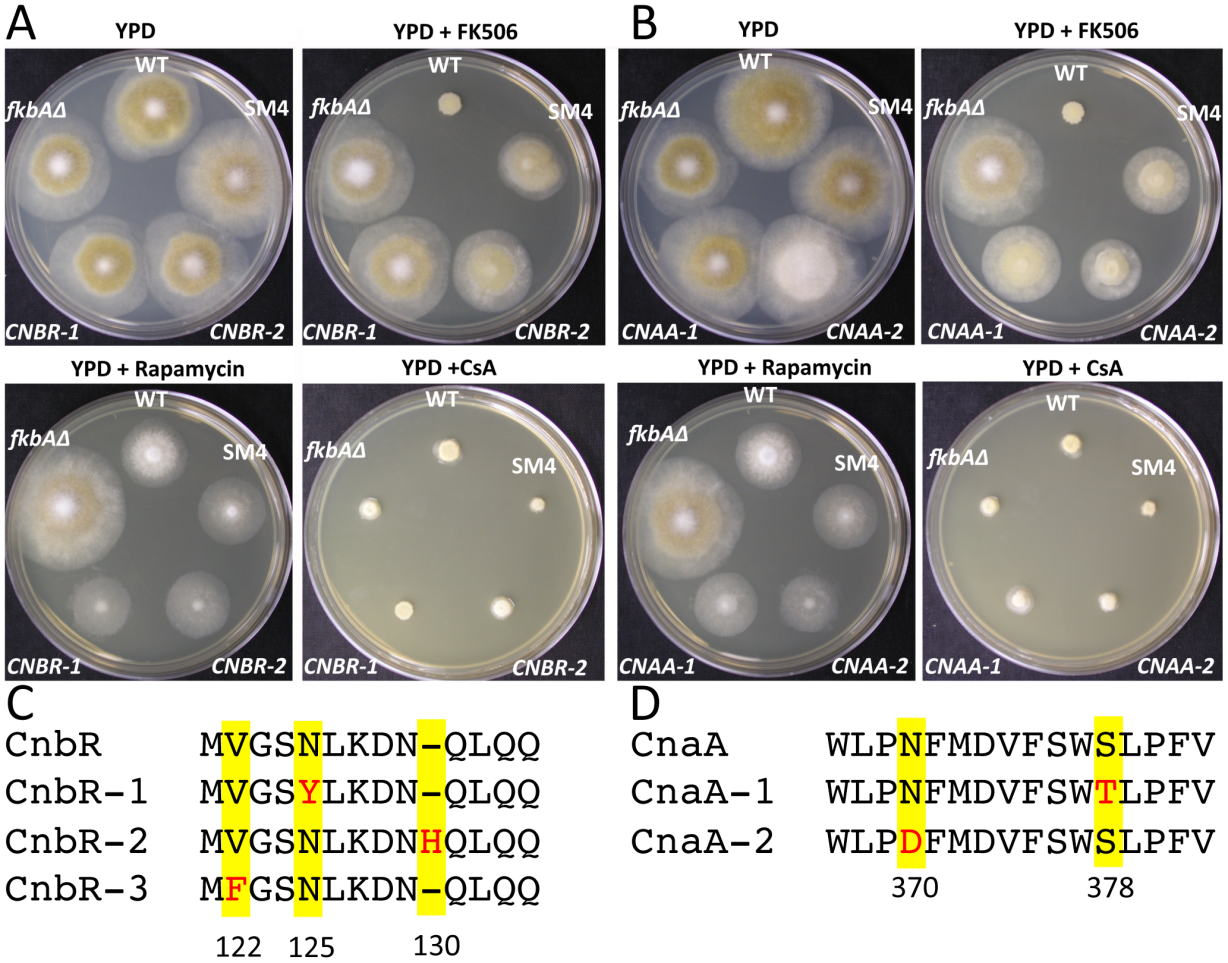
Phenotype of the spontaneous mutants that are resistant to FK506 and sensitive to rapamycin and CsA. (A and B) The four mutants MSL11 (*CNBR-1*), MSL12 (*CNBR-2*), MSL15 (*CNBR-1*), and MSL16 (*CNBR-3*) each have a mutation in the latch region of the *cnbR* gene (also see Figure S4) and the mutants formed mycelia when the calcineurin inhibitor FK506 (1 µg/mL) is present in the media (The *CNBR-3* mutant is shown in Figure S6). The *CNBR-1* and *CNBR-2*, and *CNBR-3* mutants are still sensitive to rapamycin. The *CNBR-1* and *CNBR-2* mutants remain sensitive to CsA, whereas *CNBR-3* was found to be CsA cross-resistant (Figure S5 and S6). The *fkbAΔ* mutant and L91P mutants, by contrast, are resistant to FK506 and rapamycin due to the absence or alteration of FKBP12, the target for both drugs, respectively. (C and D) The MSL13 (*CNAA-1*) and MSL14 (*CNAA-2*) mutants have mutations in the *cnaA* gene (see also Figure S7), resulting in amino acid alterations in the binding domains for calcineurin B and for the FKBP12-FK506 complex. Similarly to the *CNBR-1*, and *CNBR-2*, the *CNAA-1* and *CNAA-2* mutants form hyphae in the presence of FK506 but remain sensitive to rapamycin and CsA.

To test whether these molecular principles of FK506 sensitivity and resistance apply in *M. circinelloides*, we grew wild-type strains (10^3^ spores) in solid YPD media containing 1 µg/ml FK506 until resistant mycelial growth emerged out of yeast colonies as previously described [65]. Through this approach six FK506 resistant spontaneous mutants, MSL11, MSL12, MSL13, MSL14, MSL15, and MSL16 were generated (Table 1 and Figure 3). These FK506 resistant mutants were as rapamycin sensitive as the wild-type parental strain and in contrast to an *fkbAΔ* strain lacking FKBP12 that is FK506 and rapamycin resistant. Another mutant strain SM4 has a base substitution in the *fkbA* gene resulting in a leucine to proline substitution (L91P) in FKBP12 that confers resistance to FK506 but not to rapamycin [65]. Five of six spontaneous mutants remained sensitive to CsA, whereas the MSL16 mutant (*CNBR-3*) was found to be cross-resistant to CsA (see Figure S6). These observations led us to examine the genes involved in the mode of inhibition by FK506, and we sequenced the *cnbR*, *cnaA*, *cnaB*, and *cnaC* calcineurin genes as well as the *fkbA* gene to test if any mutations were present in any of these genes in the six FK506 resistant isolates.

**Table 1:** Strains and plasmids used in this study.

	Name	Genotypes	Remarks
	ATCC1216a	Wild-type, *M. circinelloides* f. *lusitanicus*	
	ATCC1216b	Wild-type, *M. circinelloides* f. *lusitanicus*	
	NRRL3631	Wild-type, *M. circinelloides* f. *lusitanicus*	
	NRRL1443	Wild-type, *M. circinelloides* f. *lusitanicus*	
*M. circinelloides*	ATCC1207a	Wild-type, *M. circinelloides* f. *griseocyanus*	
	ATCC1207b	Wild-type, *M. circinelloides* f. *griseocyanus*	
	CNRMA04.805	Clinical isolate, *M. circinelloides* f. *circinelloides*	
	CBS277.49	Wild-type, *M. circinelloides* f. *lusitanicus*	
	R7B	ATCCC90680, CBS277.49 background, *leuA* ^−^	
	SM4	R7B origin, L91P mutation in the *fkbA* gene	
	MU402	CBS277.49 background, *pyrG^−^ leuA^−^*	
	MU406	MU402 origin, *dcl1Δ*::*pyrG pyrG^−^ leuA^−^*	
	MU407	MU402 origin, *dcl1Δ*::*pyrG pyrG^−^ leuA^−^*	
	MU416	MU402 origin, *ago2Δ*::*pyrG pyrG^−^ leuA^−^*	
	MU420	MU402 origin, *rdrp2Δ*::*pyrG pyrG^−^ leuA^−^*	
	MSL7	MU402 origin, *cnbRΔ1*::*pyrG pyrG^−^ leuA^−^*	This study
	MSL8	MU402 origin, *cnbRΔ2*::*pyrG pyrG^−^ leuA^−^*	This study
	MSL9	MU402 origin, *cnaAΔ1*::*pyrG pyrG^−^ leuA^−^*	This study
	MSL10	MU402 origin, *cnaAΔ2*::*pyrG pyrG^−^ leuA^−^*	This study
	MSL11	R7B origin, *CNBR-1*	This study
	MSL12	MU406 origin, *CNBR-2*	This study
	MSL13	MU407 origin, *CNAA-1*	This study
	MSL14	MU420 origin, *CNAA-2*	This study
	MSL15	MU416 origin, *CNBR-1*	This study
	MSL16	MU402 origin, *CNBR-3*	This study
*R. delemar*	RA99-880	Wild-type	
*C. neoformans*	H99	Wild-type	
	pCR2.1-TOPO	PCR fragment cloning vector, Ap^R^, Kan^R^	Invitrogen
Plasmids	pCnbR-KO	*cnbRΔ* disruption allele in pCR2.1-TOPO	This study
	pAL1-1	*cnaAΔ* disruption allele in pCR2.1-TOPO	This study

Mutants in this study are all CBS277.49 (*Mucor circinelloides* f. *lusitanicus*) background. The CBS277.49 genome sequence is complete and available (see Materials and Methods).

Four strains had mutations in only the *cnbR* gene (Figure 3 and S4). The MSL11 and MSL15 mutants (*CNBR-1*) contained a point mutation, A496T, which results in an amino acid alteration, N125Y. It is interesting that two independent mutant strains contained an identical mutation in the *cnbR* gene and these strains displayed yeast growth in the presence of CsA (CsA hypersensitive) (Figure S5). This mutation may both impair FKBP12-FK506 binding and compromise the stability of the calcineurin A–B complexes to result in CsA hypersensitivity. The MSL12 strain (*CNBR-2*) contained an insertion of three nucleotides ‘CCA’ at the 513^th^ bp of the ORF (513_514insCCA), resulting in an insertion of histidine (H) after 129^th^ aspragine, Asn129_Gln130insHis. The MSL16 mutant (*CNBR-3*) contained a point mutation, G487T, which results in the amino acid substitutions, V122F. Interestingly, all of these amino acid alterations are present in the latch region of the calcineurin B regulatory subunit that is known to interact with the FKBP12-FK506 complex and to be involved in phosphatase activity [52], [66]. It is likely that the N125Y, N129_Q130insH, and V122F changes alter the interaction between FKBP12-FK506 and the calcineurin B-calcineurin A complex, resulting in resistance to FK506. Interestingly, the V122F substitution may also alter the interaction between cyclophilin-CsA and the calcineurin B-calcineurin A complex as the MSL16 mutant (*CNBR-3*, V122F) was found to be cross-resistant to CsA (Figure S6).

The two other mutant strains had mutations in only the *cnaA* gene (Figure 3 and S7). The MSL13 mutant (*CNAA-1*) has a point mutation, G1514C in the ORF, resulting in an amino acid change S378T, which lies in the amino acid adjacent to a previously known FK506 resistant mutant in calcineurin A in *S. serevisiae* (W430C) [63]. The MSL14 mutant (*CNAA-2*) has a point mutation, A1489G in the ORF, resulting in the amino acid substitution, N370D. These mutations occurred in the binding domain for calcineurin B and for the FKBP12-FK506 complex and likely reduce the binding affinity of the FKBP12-FK506 complex to the interface between the calcineurin A and B subunits, resulting in a less efficient inhibition of calcineurin activity by FK506. The fact that two FK506 resistant alleles were isolated in one of the three calcineurin A catalytic subunit (CnaA) suggests that it alone is sufficient to promote hyphal growth when cells are exposed to FK506, as the remaining two calcineurin A catalytic subunits (CnaB and CnaC) in complex with the CnbR regulatory subunit should be inhibited by FKBP12-FK506 under these conditions. The isolation and analysis of these FK506 resistant mutants in calcineurin A and B subunits further support the conclusions that the calcineurin pathway is: 1) conserved, 2) responsible for the dimorphic transition, and 3) the target of FK506 action in *M. circinelloides*.

### Disruption of the regulatory subunit gene *cnbR* results in a yeast-locked *M. circinelloides* mutant that is less virulent than wild-type

FK506 treatment results in yeast growth in *Mucor*; therefore, we hypothesized that calcineurin activity is essential to maintain hyphal growth. To test this hypothesis we disrupted the *cnbR* gene encoding the calcineurin B regulatory subunit as described in the Materials and Methods. The *cnbR* gene in the wild-type strain MU402 (*pyrG* ^−^, *leuA* ^−^) was replaced with a *cnbRΔ::pyrG* allele, and the gene deletion was confirmed by 5′ and 3′ junction PCR as well as ORF spanning PCR, and further confirmed by Southern blot analysis to show precise homologous recombination and the absence of any ectopic integration events (Figures S8 and S9). We generated two independent *cnbRΔ* deletion mutants from separate transformations.

**Figure 4:**
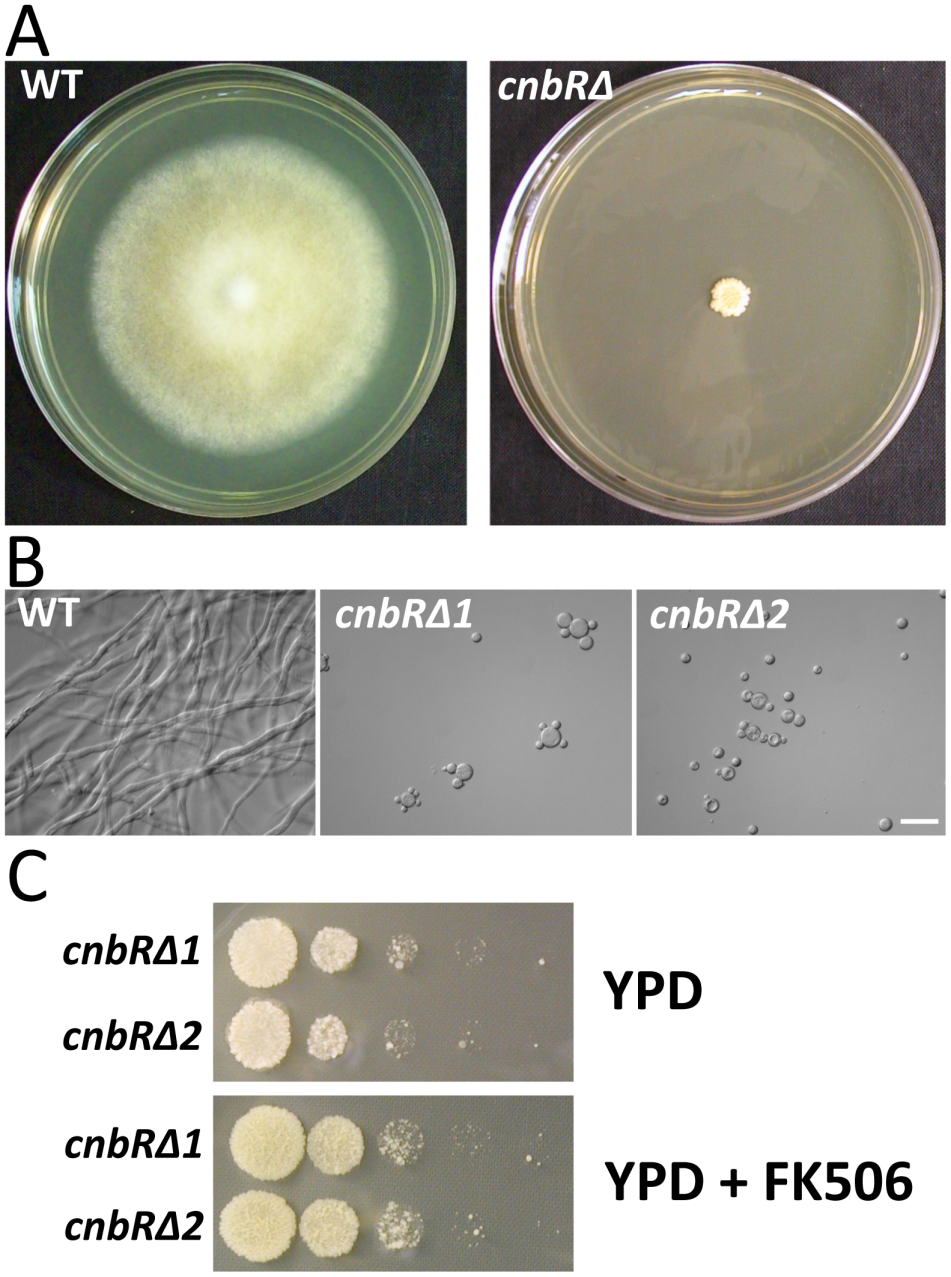
*cnbRΔ* mutants lacking the calcineurin B regulatory subunit are locked in yeast phase growth. (A) On YPD solid media, the *cnbRΔ* mutants lacking all calcineurin activity (CnbR is the common subunit partner for the three catalytic subunits CnaA, CnaB, and CnaC) produce a small compact colony; however, wild-type forms a complex mycelial mat. (B) In liquid media with vigorous shaking for aeration, wild-type grows as a filamentous/hyphal fungus, whereas the *cnbRΔ* mutants only exhibit multi-budded yeast growth as observed in wild-type grown in the presence of FK506 or anaerobic/high CO_2_ conditions. (C) The two *cnbRΔ* mutants were serially diluted and spot-inoculated on YPD or YPD with FK506 (1 µg/L). FK506 treatment did not impact growth of the mutants. Scale = 20 µm.

The wild-type and *cnbRΔ* mutants (10^3^ cells) were inoculated in the center of solid YPD medium and the strains were grown for three days at 30°C under normal aerobic conditions. The wild-type formed a large colony consisting of complex mycelia, whereas the *cnbRΔ* mutants displayed a compact yeast colony (Figure 4A). In liquid YPD media, wild-type *Mucor* grew as a filamentous mold; however, the *cnbRΔ* mutants displayed only yeast growth, even in aerobic conditions (Figure 4B). No impact of FK506 was observed on the *cnbRΔ* mutants consistent with a complete absence of the drug target (Figure 4C). This phenotype of the *cnbRΔ* mutants is essentially identical to that of wild-type *Mucor* grown in the presence of FK506 in aerobic conditions (Videos S2 and S3). The observations that mutation or inhibition of calcineurin enforces yeast growth are the key findings that allow us to conclude that the calcineurin pathway orchestrates the dimorphic switch in *Mucor*.

**Figure 5:**
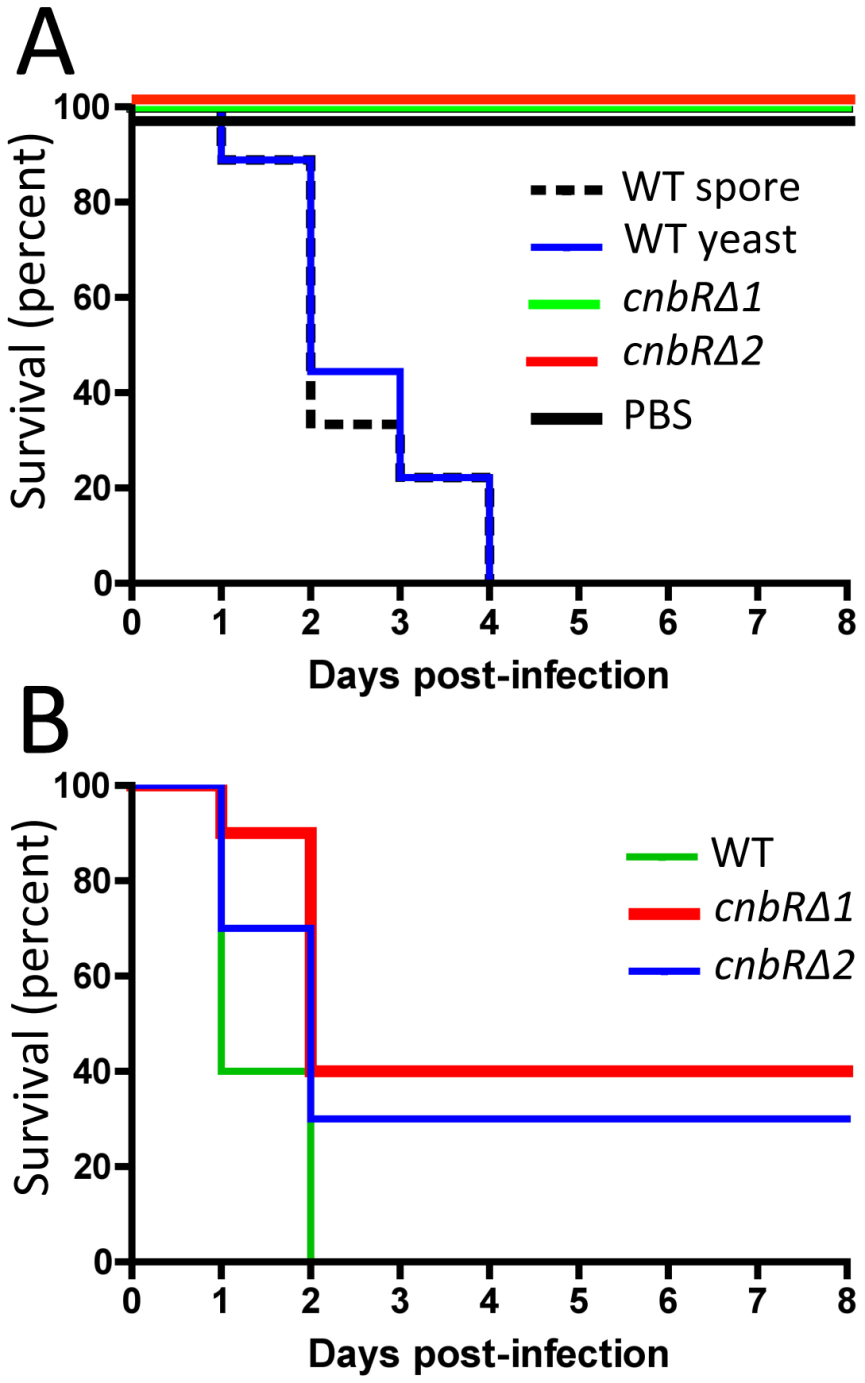
Yeast-locked mutants are attenuated in virulence in a heterologous host model. (A) 20,000 wild-type spores, wild-type yeast, or the *cnbRΔ* yeast-locked mutants were inoculated into wax moth larvae. Infection with wild-type spores or yeast resulted in 100% mortality by day 4 post-infection; however, the *cnbRΔ* yeast-locked mutants were significantly less virulent compared to wild-type spores or yeast cells. (B) At an inoculum of 40,000 cells, the *cnbRΔ* mutants exhibited 60 to 70% mortality by day 2 post-infection, whereas wild-type spores showed 100% mortality.

To assess the virulence of yeast vs. hyphae and to test the role of the calcineurin pathway in virulence, the *cnbRΔ* yeast-locked mutants were compared to both wild-type spores and wild-type yeast cells in animal virulence models (Figure 5). The R7B (*leuA^−^*) strain served as wild-type to exclude the possibility that leucine auxotrophy could generate a biased result, as the *cnbRΔ* mutants were generated in the MU402 (*pyrG^−^ leuA^−^*) strain background (*pyrG^−^ leuA^−^ cnbR::pyrG*). Wild-type spores, wild-type yeast, and *cnbRΔ* mutant yeast (two independent mutant isolates) were quantified and cohorts of 10 wax moth larvae (a heterologous host system) per strain were infected with an inoculum of 20,000 infectious units. Wild-type spores and wild-type yeast both showed 100% mortality by four days post-infection (Figure 5A). Wild-type yeast are able to switch into growth as hyphae; thus, it was anticipated that wild-type yeast would be as virulent as wild-type spores. On the other hand, the *cnbRΔ* yeast-locked mutants did not kill the larvae after 8 days and were indistinguishable from the PBS control. When 40,000 cells were inoculated, the yeast-locked *cnbRΔ* mutants showed a higher level of virulence (60 to 70% mortality by day 4 post-infection), but were still significantly less virulent than wild-type spores (WT vs. *cnbRΔ1*, p = 0.00059) (Figure 5B). These results indicate that: 1) morphogenesis contributes to virulence in this fungus; 2) the calcineurin pathway is involved in virulence, and 3) calcineurin inhibitors have promise as antifungal drugs for zygomycete infections.

That hyphae are more virulent than yeast, or alternatively that ability to transition between yeast to hyphae is a key virulence factor as observed in *Candida albicans* [40], is further supported by observations of tissues from infected animal host models (Figure S10). In a wax moth mucormycosis system, wild-type mainly presented as the hyphal form, whereas the yeast-locked mutants were exclusively as yeast. In a murine mucormycosis system, we found that the majority of an *M. circinelloides* fungal burden was present in the brain as hyphae.

### cAMP-dependent protein kinase A activity is elevated during yeast growth in anaerobic/high CO_2_ conditions, in the presence of FK506, and in the *cnbRΔ* mutants

**Figure 6:**
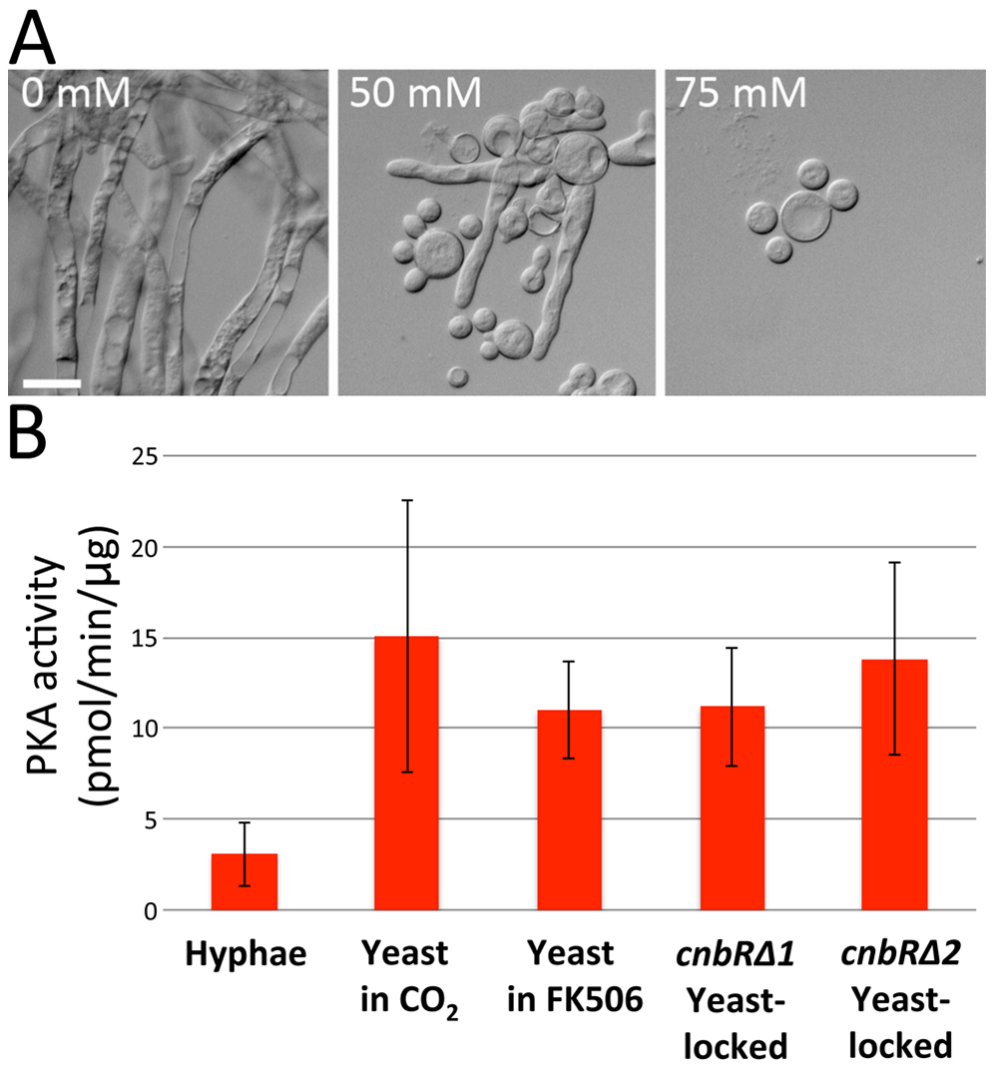
Protein kinase A activity is elevated during yeast growth. (A) Bicarbonate ions (HCO_3_^−^) that are equivalent to a high concentration of CO_2_ induce yeast growth of *Mucor*. Scale = 20 µm. (B) Crude protein extract (0.5 µg) from each sample was subjected to a PKA-specific kinase activity measurement with the SignaTECT cAMP-Dependent Protein Kinase (PKA) Assay System (Promega, Madison, WI). Protein kinase A activity was elevated during yeast growth phase induced by CO_2_, and similar results were observed during yeast growth enforced by FK506 or in the *cnbRΔ* yeast-locked mutants, indicating that higher PKA activity is correlated with yeast growth and calcineurin may negatively regulate PKA activity. Error bars represent the standard deviation of the mean from three independent assays (from left to right, the values are: 3.06±3.01; 15.04±12.99; 11±4.6; 13.82±9.16).

Yeast growth can be induced by elevated levels of CO_2_ [15], [16]. Carbon dioxide is converted into bicarbonate ions (HCO_3_^−^) by carbonic anhydrase (reviewed in [67] and references therein). Subsequent activation of adenylyl cyclase by CO_2_, HCO_3_^−^, or both occurs, resulting in the production of cAMP. cAMP then binds to the regulatory subunit of protein kinase A (PKAR) and the protein kinase A (PKA) catalytic subunits are released to result in activation. Previous studies suggested that PKA may play a major role during yeast growth, given that the PKA genes are over-expressed during yeast growth, and the regulatory subunit gene (*pkaR*) that inhibits PKA activity is expressed at a higher level during the yeast to hyphal transition [25], [27], [28]. We further confirmed that *Mucor* grows as yeast in the presence of high concentrations of bicarbonate in the media. At 50 mM bicarbonate, *Mucor* exhibited both hyphal and yeast growth; at 75 mM, *Mucor* grew only as a yeast (Figure 6A).

We adapted a PKA activity assay used in other studies of *Mucor* morphogenesis to test how PKA activity is differentially regulated during yeast and hyphal growth [27], [28], [68]. Due to the nature of this assay performed in the presence of cAMP, the maximum possible PKA activity was measured. Crude cell extracts (0.5 µg total protein) were prepared and PKA activity was measured by using the PKA specific substrate kemptide as previously described [27], [68]. Compared to hyphal growth, PKA activity was elevated approximately five-fold during yeast growth in high CO_2_ growth conditions (Figure 6B). During yeast growth enforced by FK506, PKA activity was higher. In the two independent *cnbRΔ* yeast-locked mutants, PKA activity was also higher than in wild-type (Figure 6B). These results demonstrate that 1) higher activity of PKA is associated with, and may be necessary for, yeast growth; and 2) calcineurin and PKA may play antagonistic roles during the yeast-hyphal dimorphic transition. It is noteworthy that the *M. circinelloides* genome encodes four PKA regulatory subunits and their subcellular localization may generate compartmentalized PKA activity [26]–[28]. In the non-dimorphic zygomycete *R. delemar*, PKA activity was also elevated by FK506 treatment, and the same result was observed in the basidiomycete pathogen *C. neoformans* (Figure S11).

### Calcineurin catalytic A subunits are expressed differentially during hyphal and yeast growth

We tested whether the three catalytic A subunit genes (*cnaA*, *cnaB*, and *cnaC*) are differentially regulated during the dimorphic transition. Initial northern blots showed that the probes for each *cna* gene were cross-reactive due to sequence similarity (data not shown); therefore, we performed quantitative RT-PCR with gene specific primers to specifically examine expression of each individual gene. In RT-PCR analyses, we found that all three *cna* genes were expressed, indicating that none were pseudogenes (data not shown). The PCR products from cDNAs obtained for each gene were sequenced to re-annotate the genes. Based on the ORF sequences, we designed a pair of specific RT-PCR primers across two exons of each *cna* gene. The actin gene was used for normalization.

**Figure 7:**
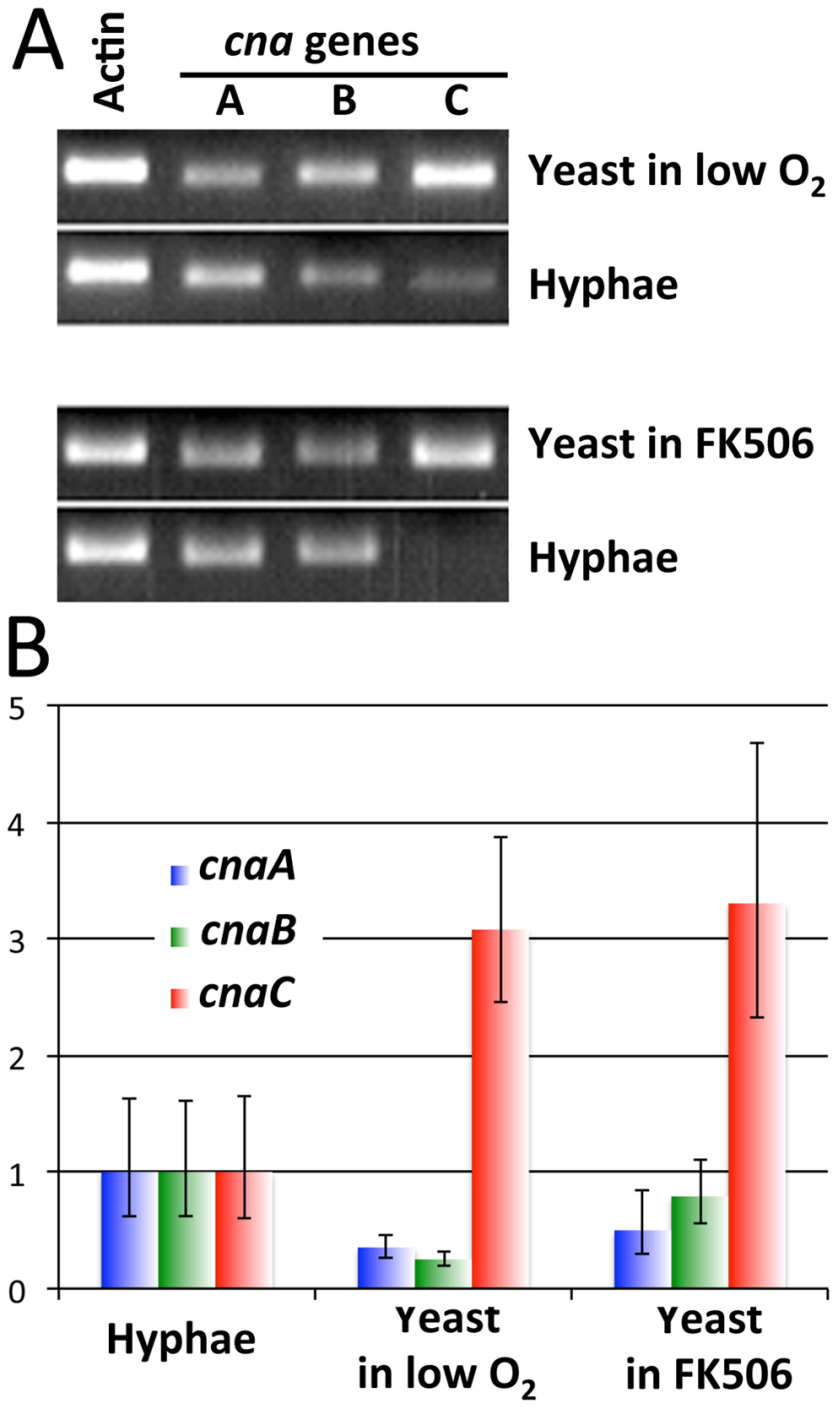
Three *cna* genes in *M.* *circinelloides* are differentially expressed. (A) The three *cna* gene are differentially expressed during yeast and hyphal growth. A difference in intensity of the *cnaC* gene RT-PCR product was apparent when yeast induced by low oxygen/high CO_2_ or FK506 were compared with hyphae. (B) Expression of the *cnaC* gene increased ∼3-fold during yeast growth compared to hyphal growth; however, expression of both *cnaA* and *cnaB* decreased during yeast growth based on qRT-PCR analyses. The actin gene served as a normalization control, and the hyphal growth stage was used as a control to measure the expression of the *cna* genes during yeast growth.

The expression levels of the *cna* genes were evaluated using ct values from RT-PCR with hyphal growth conditions as a control. We found that *cnaC* is expressed at higher levels during yeast growth under microaerobic conditions but at lower levels during hyphal growth. Interestingly, during yeast growth driven by FK506, *cnaC* is also overexpressed (Figure 7). The expression levels of *cnaA* and *cnaB* were moderately reduced during the dimorphic switch. These results suggest that CnaC plays a specific role during the yeast growth phase and could therefore be less sensitive to inhibition by FK506, possibly as the result of overexpression in molar excess above FKBP12 levels. In parallel studies, Drs. Praveen Juvvadi and William Steinbach at Duke University found that *M. circinelloides cnaC* partially complements the radial growth defect of the *A. fumigatus* calcineurin catalytic A subunit (*cnaA*) mutant and also that MuCnaC-GFP localizes to septa, as does AfCnaA (Juvvadi P. R. et al., manuscript in preparation). *M. circinelloides* hyphae are coenocytic (aseptate), but during yeast growth or pseudohyphal growth, septa are formed; thus, CnaC function may be associated with septum formation.

### Disruption of the *cnaA* gene results in a hypersensitivity to calcineurin inhibitors and SDS, the production of larger spores, and a loss of hyphal polarity

Of the three *cna* genes found in *Mucor*, homologs for the *cnaA* gene were found in all three of the zygomycete genomes analyzed (Figure S3), indicating that the *cnaA* gene may be the more ancestral calcineurin catalytic A subunit gene. Therefore, we chose to disrupt the *cnaA* gene to further test the roles of calcineurin in *Mucor*. A disruption allele consisting of the *pyrG* gene flanked by ∼1 kb of the 5′ upstream and 3′ downstream untranslated regions of the *cnaA* gene were introduced as described in the Materials and Methods. Gene replacement by recombination was confirmed by 5′ and 3′ junction PCR, ORF spanning PCR, and Southern blot (Figures S12 and S13). Two independent *cnaAΔ::pyrG* mutants were obtained from separate transformations.

**Figure 8:**
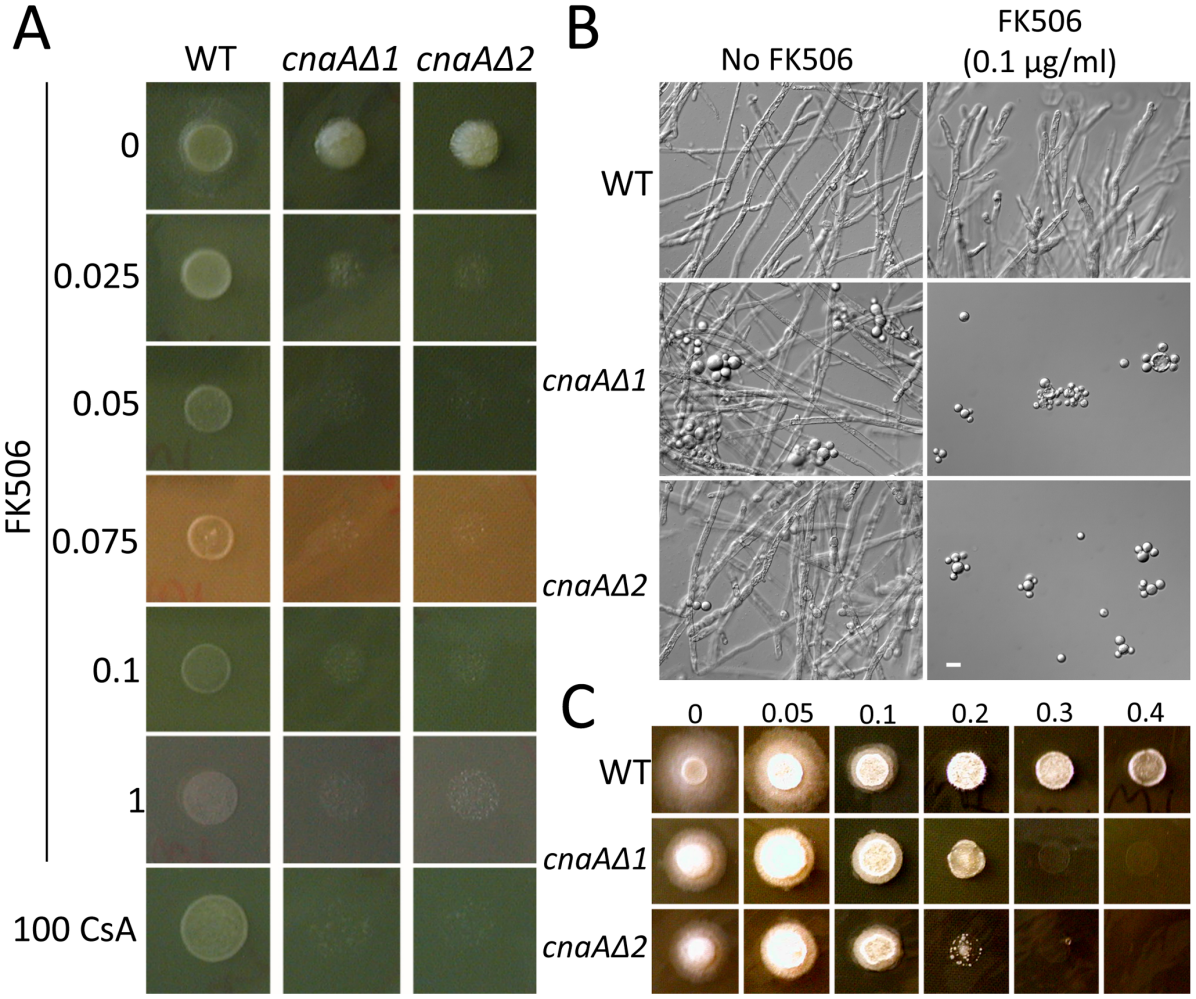
*cnaAΔ* mutants are sensitive to calcineurin inhibitors and SDS. (A) Two independently derived *cnaA* mutants exhibit hypersensitivity to FK506 or CsA. In the presence of low concentrations of FK506 (0.1 µg/L), growth of the *cnaAΔ* mutants was significantly impaired compared to the wild-type, which formed a readily visible colony after only 1 day. (B) In liquid culture with a low concentration of FK506 (0.1 µg/mL), at which wild-type grows as hyphae, the *cnaAΔ* mutants grew as multi-budded yeast cells. Even in culture without FK506, the *cnaAΔ* mutants exhibited a mixture of yeast and hyphae compared to wild-type, which was exclusively hyphal. Scale = 20 µm. (C) The *cnaAΔ* mutants were more sensitive to SDS in YPD media than wild-type, suggesting that the *cnaAΔ* mutants fail to have compromised cell wall integrity. The percentage concentration (v/v) of SDS is displayed in the panel.

Interestingly, the *cnaAΔ* mutants exhibited much higher sensitivity to FK506 and CsA compared to wild-type (Figure 8A). Sensitivity was assessed based on the ability of the drugs to inhibit growth of the strains tested; for example, on YPD medium supplemented with FK506 (0.025 to 1 µg/ml), the two *cnaAΔ* mutants displayed significantly reduced growth compared to wild-type. Both *cnaAΔ* mutants also exhibited hypersensitivity to CsA. In liquid YPD with vigorous aeration, more abundant yeast growth was observed in the *cnaA* mutants compared to wild-type. Notably, the *cnaAΔ* mutants exhibited only yeast growth in the presence of 0.1 µg/ml FK506 in the media, which is a concentration insufficient to inhibit hyphal growth and induce yeast growth in wild-type (Figure 8B). We explain these observations as resulting from the absence of one catalytic A subunit out of the three expressed (the mutants still have intact *cnaB* and *cnaC* genes), leading to a relative decrease in calcineurin, similar to haplo-insufficiency in *S. cerevisiae* [69]. The *cnaAΔ* mutants were also more sensitive to SDS, indicating that calcineurin may be involved in cell wall integrity (Figure 8C) as observed in *Candida* species [57], [70]. *Mucor* was largely resistant to Congo red and calcofluor white and there was no significant difference between wild-type and *cnaA* mutants with either (data not shown).

**Figure 9:**
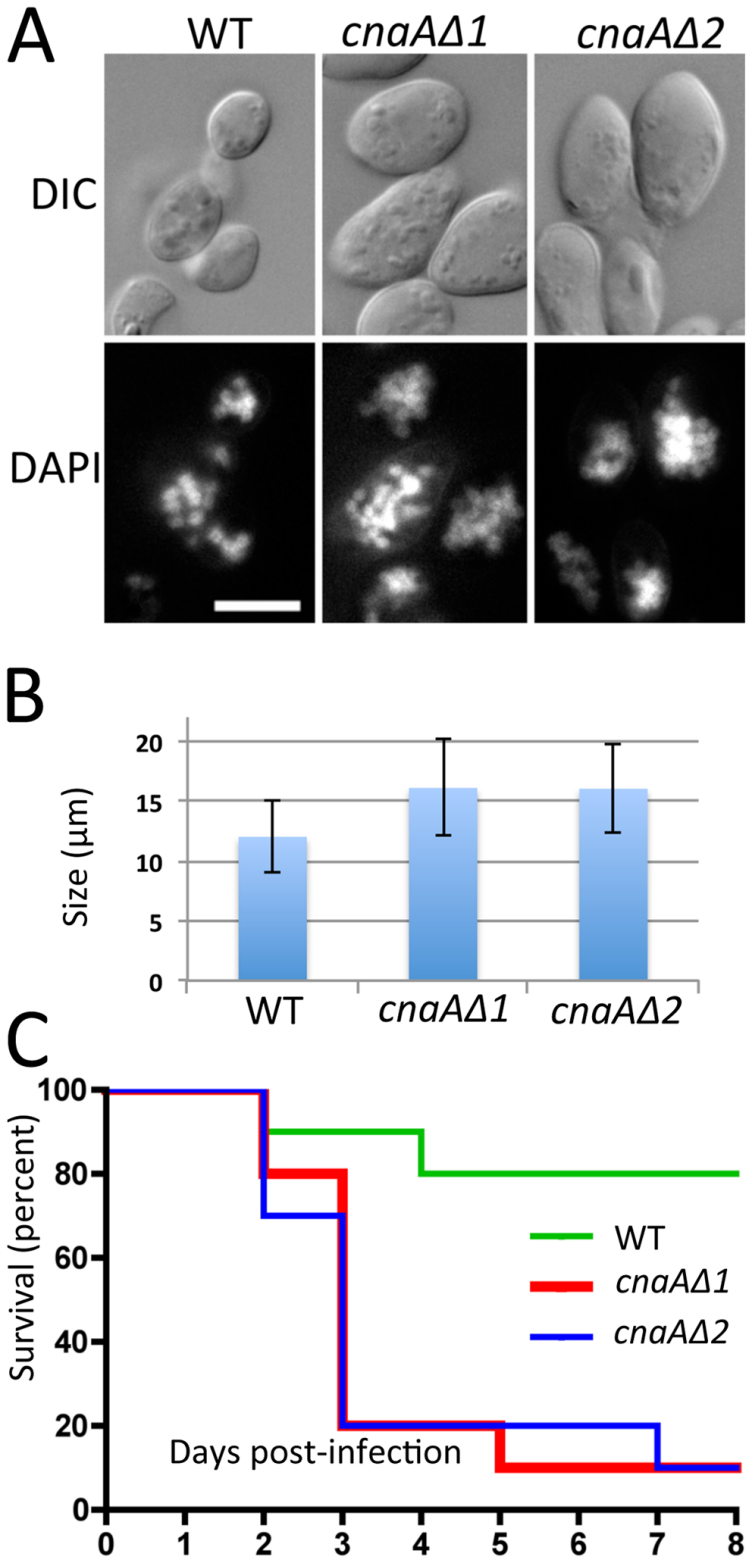
*cnaAΔ* calcineurin A subunit mutants produce more virulent and larger spores than wild-type. (A) The *cnaAΔ* mutants and wild-type produced multinucleate spores (stained with DAPI), and the mutant spores were larger than those of wild-type. DIC: differential interference contrast, DAPI: nuclei. (B) Wild-type produced spores with a size of 12.0±2.95 µm, whereas the *cnaAΔ1* and *cnaAΔ2* strains produced significantly larger spores with a size of 16.1±4.04 or 16.0±3.70 µm, respectively. The difference in spore size between wild-type and the *cnaAΔ* mutants was statistically significant (p<0.0001, N = 80). (C) The larger spores of the *cnaAΔ* mutants were more virulent than wild-type at the inocula tested (N = 5,000), suggesting that calcineurin is involved in spore size determination and providing further evidence that spore size is linked to the virulence of this fungus.

We found that the *cnaAΔ* mutants produce larger spores than the wild-type (WT: 12.00±2.94 µm; *cnaAΔ1*: 16.08±4.04 µm; *cnaAΔ2*: 16.02±3.70 µm) (Figure 9A and B). The differences in spore size between the wild-type and the two *cnaAΔ* mutants are significant based on a two sample independent t-test (p<0.0001 in both WT vs. *cnaAΔ1* and WT vs. *cnaAΔ2*, N = 80). The *cnaA* mutant spores were multinucleate as are wild-type spores. This larger spore size is an intriguing phenotype because spore size is a virulence factor as shown in our previous study, wherein larger spores were more virulent than smaller spores [53].

To test if the enlarged spore phenotype conferred by mutations in the *cnaA* gene might contribute to virulence, we infected wax moth larvae with 5,000 spores of wild-type or the *cnaAΔ* mutants. Ten larvae were used for each strain. At the given inoculum, wild-type showed a moderate level of virulence; however, the two *cnaA* mutants were significantly more virulent compared to the wild-type (Figure 9C). The *cnaAΔ* mutants also formed hyphae in hosts similar to wild-type. (Figure S14). The experiments were performed independently three times with similar results. As observed in our previous study [53], in the interaction with macrophages, the larger *cnaAΔ* mutant spores produced a germ tube inside of macrophages, whereas wild-type remained as spores without germ tube emergence at 3.5 hours of co-culture (Figure S15), indicating that the mutants possibly avoided the innate host immune system by sending out germ tubes earlier and more efficiently than the wild-type. This suggests that calcineurin is involved in spore size control as a negative regulator and may therefore also contribute to virulence, although other calcineurin-CnaA-dependent functions may also contribute.

**Figure 10:**
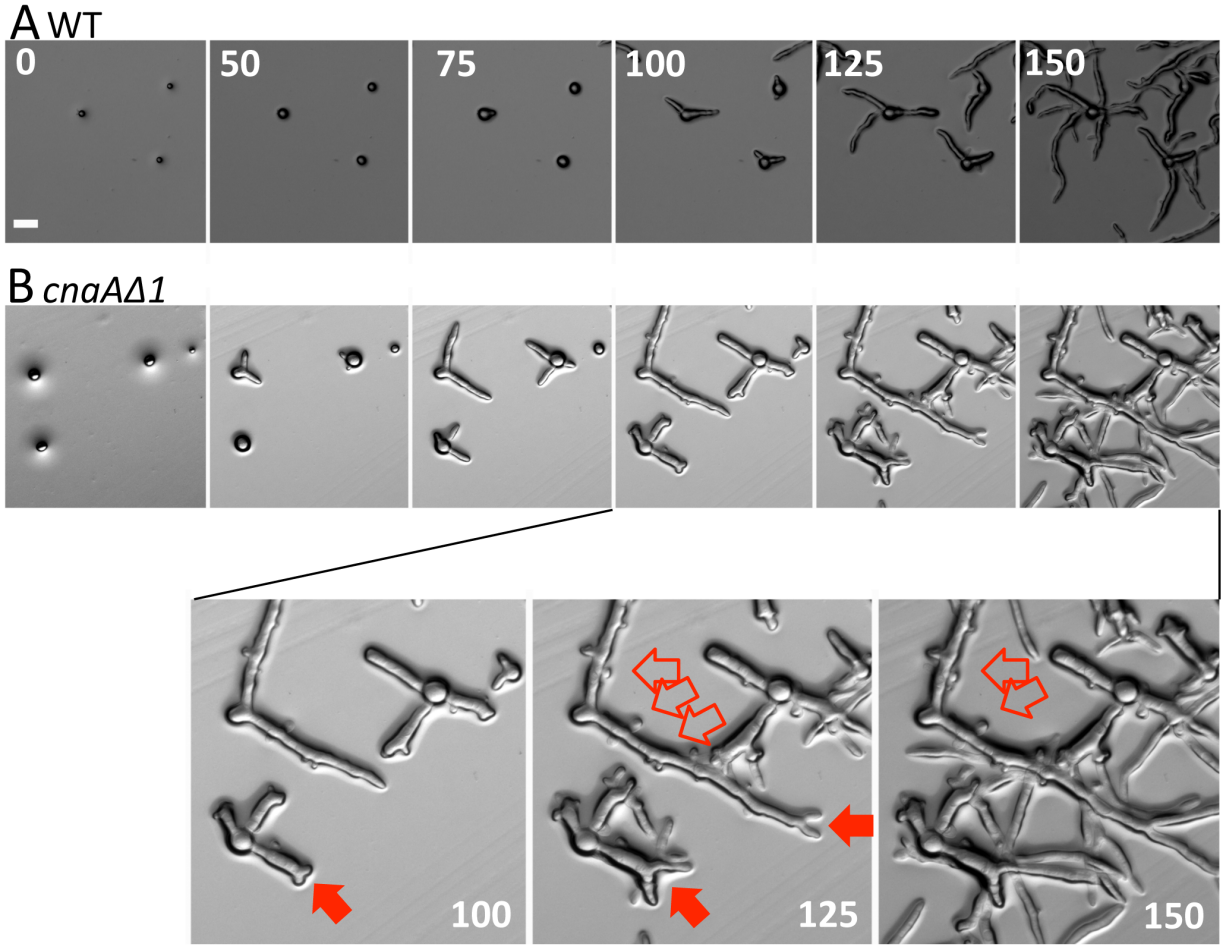
Hyphal growth is altered in *cnaAΔ* mutants. (A) Wild-type spores first grow isotropically and then polarity is established to initiate germ tube emergence. During hyphal growth, polarity is maintained properly in order to grow directionally. (B) Compared to wild-type, the *cnaAΔ* mutants exhibited a shorter isotropic growth phase due to their larger initial spore size (the *cnaAΔ* mutants germinated at 50 minutes after inoculation, whereas wild-type started germination at 75 minutes after inoculation, as shown in the figure) [53]. *cnaAΔ* mutant hyphae undergo tip-splitting (solid red arrows), indicating that hyphal polarity is not properly maintained during mycelial growth. The *cnaAΔ* mutants also show an abnormal hyphal branching pattern (open red arrows). The numbers on the figure indicate the time (in minutes) after inoculation. Scale = 20 µm.

The *cnaAΔ* mutations also confer abnormal hyphal polarity. To carefully examine hyphal growth of the *cnaAΔ* mutants, we applied a time course imaging technique with a microscope equipped with an automatic shutter system. Wild-type and *cnaAΔ* mutant spores were placed on YPD agar media, and images were captured every 30 seconds. When the *cnaAΔ* mutants germinated, the germ tubes exhibited a tip-splitting phenotype and abnormal branching, whereas wild-type germ tubes elongated in a single direction with no tip-splitting (Figure 10 and Videos S4 and S5). This phenotype of the *cnaAΔ* mutants indicates that CnaA is necessary to maintain a single hyphal polarity in *Mucor*. A role for calcineurin in hyphal polarity is conserved in other filamentous fungi, including *Neurospora crassa* and *A. fumigatus* [62], [71], indicating that this is a general role of calcineurin.

## Discussion

Morphogenic transitions (for example, yeast to hyphae and vice versa) are common in fungi and evolved in multiple fungal lineages. Many well known fungal pathogens change their morphology in response to their environments and morphogenesis is linked to their virulence. Human fungal pathogens encompass ascomycetes, basidiomycetes, and zygomycetes [72]. In ascomycetes and basidiomycetes, the relationships between dimorphism and pathogenicity are relatively well established. However, whether dimorphism is associated with virulence in zygomycetes was not known. Our study has revealed how the dimorphic transition is regulated and linked to virulence and how the calcineurin pathway directs this process in the human pathogenic zygomycete *M. circinelloides*.

Several zygomycete species, especially those belonging to the order Mucorales, are known to cause the lethal fungal infection mucormycosis. These species include *Mucor* spp., *Rhizopus* spp., *Rhizomucor* spp., *Cunninghamella* spp., and *A. trapeziformis* [1]–[3]. Unlike ascomycetous and basidiomycetous fungal pathogens, the current status of research into zygomycete pathogens is in its infancy. A limited ability to conduct genetics studies is one of the barriers to advancement in our understanding of zygomycete pathogens. *M. circinelloides* is one of the best developed systems for genetics and molecular biology among zygomycete species. Therefore, *Mucor* provides a foundation from which to understand mucormycosis.

*Mucor* is a dimorphic fungus and the morphogenic transition is known to occur in response to environmental conditions, especially low levels of oxygen and high levels of carbon dioxide. Several other studies have implicated protein kinase A in this response [25]–[31]. Other factors known to be involved predominantly alter mitochondrial functions. Our study revealed that the calcineurin pathway is a key regulator of this developmental process.

FK506 treatment imposes yeast growth in *Mucor* (Figure 1). Previously, we found that FKBP12 mutants are resistant to FK506 (resistance is defined as strains forming hyphae instead of yeast in the presence of FK506) [65]. FKBP12 is a member of the immunophilin family of proteins, which have *cis-trans* peptidyl- prolyl isomerase activity [64]. When bound to FK506, FKBP12 binds to the interface between the calcineurin catalytic A and regulatory B subunits, inhibiting phosphatase activity by occluding substrate access to the active site [51]. FKBP12 also binds to rapamycin to inhibit the Tor pathway. In other fungi, disruption of the gene encoding FKBP12 confers resistance to FK506 and rapamycin (in the absence of FKBP12, FK506 fails to bind calcineurin). Similar results are observed in *Mucor*; a splice site mutant (adenine into guanine at the 316^th^ nucleotide), Leu91Pro substitution mutant, and FKBP12Δ null mutants are all resistant to FK506 [65].

In this study, we found that the N125Y, N129_Q130insH, and V122F alterations in the latch region of the calcineurin B regulatory protein confer resistance to FK506 (Figure 3 and S4). A previous site-directed mutagenesis study documented that the latch region is essential for immunophilin-immunosuppressant complex docking [52]. These mutations in the CnbR subunit of *Mucor* may prevent or limit docking of FKBP12-FK506 onto the calcineurin complex to result in less inhibition of calcineurin. Similar results has been observed in *C. neoformans* where a two amino acid insertion in the latch area of calcineurin B similarly confers resistance to FK506 [66]. In addition, the N370D and S378T mutations in the calcineurin B and FKBP12-FK506 binding domain of the CnaA catalytic subunit (Figure 3 and S7) confer resistance to FK506 and lead to the conclusion that *Mucor* has a conserved calcineurin pathway. Furthermore, the calcineurin B regulatory subunit gene disruption mutants only exhibited yeast growth, even during vigorous aeration conditions and there was no impact of FK506 on these strains that lack calcineurin (Figure 4). These observations all support the conclusion that calcineurin is a key factor in the dimorphic yeast to hyphae transition in *Mucor*.

Calcineurin has been suggested as a candidate antifungal drug target in many other pathogenic fungi, including *Candida* spp., *C. neoformans*, *A. fumigatus*, *Magnaporthe oryzae*, and *U. maydis* (reviewed in [55]). The observation that calcineurin inhibitors block hyphal growth in zygomycete pathogens parallels findings in other fungal pathogens. That transplant patients receiving FK506 treatment have a lower incidence of mucormycosis contributes to validate calcineurin as a potential antifungal drug target [73]. FK506 displays synergistic effects when combined with other antifungal agents, including azoles and echinocandins in *C. albicans* (reviewed in [74]). In addition, calcineurin inhibitors exhibit an *in vitro* synergistic inhibition on zygomycete growth [75]–[77]. A recent study suggests that combination therapy with posaconazole and FK506 is efficacious in both a *Drosophila* heterologous host model and a cutaneous mucormycosis murine model system [78]. We found that yeast-locked *cnbRΔ* mutants are not as virulent as wild-type in a heterologous wax moth model system (Figure 5). One possibility is that hyphae are a more virulent form of *Mucor* compared to yeast. However, although the wild-type existed as hyphae and the yeast-locked mutants existed as yeast inside hosts (Figure S10), we cannot rule out the possibility that the ability to transition between yeast and hyphae is a major factor in virulence during host infections as observed in *C. albicans* [40]. Currently there are no known hyphal-locked mutants in *Mucor* that could be used to test these hypotheses further. This observation overall supports two important conclusions: first, dimorphism is linked to virulence in this fungal species and second, calcineurin inhibitors may be an effective way to control mucormycosis.

However, because the calcineurin pathway is conserved throughout eukaryotes, including humans, calcineurin inhibitors have their own inherent risks as antifungal drugs, including for example host immune suppression. In humans, calcineurin dephosphorylates the cytoplasmic subunit of the transcription factor nuclear factor of activated T-cells (NFATc) and promotes its nuclear localization to induce interleukin 2 (IL-2) and stimulate T cell proliferation [79]. Thus, calcineurin inhibitors are immunosuppressive drugs. It is possible that the effects of calcineurin inhibitors as immunosuppressants will overpower their antifungal activities. This situation was also observed in our murine host experiments, in which FK506 treatment detrimentally affected mice infected with *Mucor* (Figure S16). Therefore ideal calcineurin inhibitors would be FK506 analogs that are less immunosuppressive to the host but which remain active against fungal calcineurins.

*Mucor* encodes three calcineurin catalytic A subunits (Figures 2 and S3). The presence of the triplicated *cna* genes might have been derived from individual gene duplication events, which is supported by phylogenetic analyses and the presence of repetitive sequences linked to the *cna* genes (Figures S2 and S17). However, in *Mucor*, synteny around the three *cna* genes was not conserved (Figure S17), indicating that the duplication of the *cna* genes may have resulted from individual gene duplication events or segmental gene duplications that occurred earlier and that flanking synteny has decayed with evolutionary time. The presence of the same repetitive elements up- and downstream of the *cna* genes supports the hypothesis that individual gene duplication events may have produced the three versions of the *cna* genes in *Mucor*. However, in *R. delemar*, the triplicated copies of the *cna* genes arose via a different trajectory, involving a recent whole genome duplication and then a segmental gene duplication. Interestingly, the non-pathogenic Mucorales species *Phycomyces* has only a single *cna* gene (Figure S3). These observations indicate that different evolutionary trajectories gave rise to genes linked to virulence during speciation in the Mucorales order.

The *cnaA* gene is conserved in the three Mucorales species, and this gene might therefore have a conserved ancestral role in this fungal lineage. The *Mucor cnaAΔ* mutants display several interesting phenotypes: an abnormal polarity during hyphal growth, larger spore production, hypersensitivity to calcineurin inhibitors, and cell wall defects (Figures 8, 9, and 10, and Videos S3 and S4). The abnormal polarity phenotypes include tip-splitting and atypical branching patterns. In several other fungal species, calcineurin is involved in hyphal polarity in accord with our observations in *Mucor* [62], [71], [80]. A remarkable phenotype of the *cnaAΔ* mutants is the production of larger spores (Figure 9). In a previous study, we showed that larger spore size is linked to higher virulence in *Mucor*. Accordingly, the larger spores of the *cnaAΔ* mutants were also found to be more virulent in the heterologous host model and macrophage model (Figures 9 and S15), further supporting the hypothesis that fungal cell gigantism is involved in virulence as observed in titan/giant cells of *C. neoformans* and multinucleate spherules of *Coccidioides immitis*/*posadasii* [53], [81]–[84]. This observation is at one level paradoxical because we suggest calcineurin as a target of antifungal drugs. However, we found that the growth of the *cnaAΔ* mutants was more sensitive to FK506 and CsA, and that the *cnaAΔ* mutants also exhibit cell wall defects (Figure 8). Taken together with the finding that *cnbRΔ* mutants that should lack all calcineurin activity are attenuated in virulence, these results indicate that calcineurin inhibitors remain attractive antifungal drug candidates.

**Figure 11:**
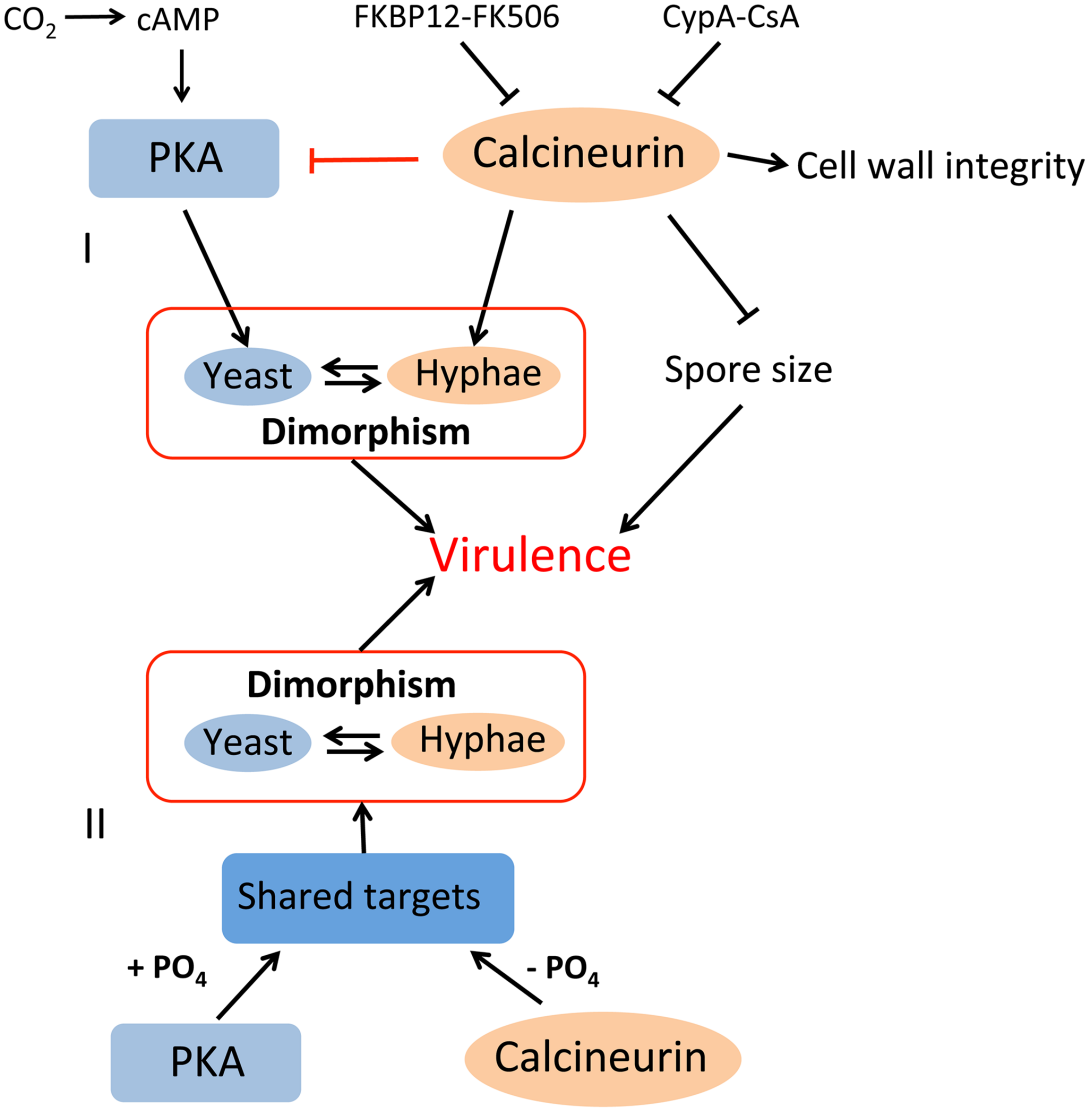
Illustration of roles of calcineurin in dimorphism and virulence of *Mucor*. Calcineurin is necessary for hyphal growth and negatively regulates spore size. Calcineurin is also involved in cell wall integrity. Calcineurin activity is inhibited by two immunophilin-drug complexes, FKBP12-FK506 and CypA-CsA, resulting in yeast growth or abnormal hyphal growth, respectively. On the other hand, during yeast growth, cAMP-dependent protein kinase A (PKA) activity is increased. I) Inhibition of calcineurin activity by FK506 or mutation of the regulatory B subunit results in elevated PKA activity, indicating that calcineurin negatively regulates PKA activity, either via direct inhibition of PKA or indirectly through other factor(s). II) Alternatively, PKA and calcineurin may share common target proteins and function. FKBP12: FK506 binding protein 12, CypA: cyclophilin A.

Calcineurin regulates many cellular processes in eukaryotes, including T-cell activation, polarized growth of neurites, long-term memory transitions, signaling in cardiac hypertrophy in humans as well as morphogenesis and virulence in fungi [55], [79], [85]–[87]; however, to our knowledge, a connection between respiration and calcineurin has not been previously described. Our finding that calcineurin negatively regulates protein kinase A (PKA) activity is also novel (Figures 6 and S11). An antagonistic relationship between calcineurin and PKA has been observed in *U. maydis* and *S. cerevisiae* [88], [89]. Our study verifies that calcineurin activity is required for hyphal growth and PKA activity is correlated with yeast growth. Further, calcineurin activity might attenuate PKA activity in *Mucor*. Similar results were observed in two other pathogenic fungi, *C. neoformans* and *R. delemar*; thus, this antagonistic interconnection between calcineurin and PKA may be a conserved functional circuit in several fungal species. It is not clear if calcineurin directly acts to dephosphorylate PKA to control its activity or indirectly regulates PKA activity, for example, by regulation at the transcriptional level or via other proteins. Alternatively, calcineurin and PKA may share common targets (e.g. Crz1) for dephosphorylation and phosphorylation, respectively (Figure 11). Future investigation of this signaling network will be a promising venture.

In conclusion, our finding that calcineurin orchestrates the dimorphic transition in *Mucor* is a significant advance since the first finding that CO_2_/low oxygen was the key factor [15]–[17]. Another key major advance of this study is that dimorphism is linked to virulence in the zygomycete pathogen *Mucor*. This study on morphogenesis and pathogenicity in the emerging zygomycete fungal pathogens will foster development of anti-mucormycosis targets for novel treatment options.

## Materials and Methods

### Ethics statement

The animal studies at the Duke University Medical Center were in full compliance with all of the guidelines of the Duke University Medical Center Institutional Animal Care and Use Committee (IACUC) and in full compliance with the United States Animal Welfare Act (Public Law 98–198). The Duke University Medical Center IACUC approved all of the vertebrate studies under protocol number A061-12-03. The studies were conducted in the Division of Laboratory Animal Resources (DLAR) facilities that are accredited by the Association for Assessment and Accreditation of Laboratory Animal Care (AAALAC).

### Strains and growth conditions

Strains and plasmids used in this study are listed in Table 1. *M. circinelloides* strains were grown on yeast and dextrose agar (YPD, 10 g/L yeast extract, 20 g/L peptone, 20 g/L dextrose, 2% agar) or yeast peptone glucose (YPG, 3 g/L yeast extract, 10 g/L peptone, 20 g/L glucose, 2% agar, pH = 4.5) media for spore production at room temperature (24°C) or at 30°C in the light. YPD was used for liquid culture with vigorous shaking for aeration at 30°C. For anaerobic/high CO_2_ conditions, 500 mL flasks were completely filled with liquid YPD media that had been sonicated to eliminate residual O_2_ gas. The spores of experimental strains (10^7^ spores) were then inoculated on the bottom of the flasks. The flasks were sealed with parafilm and placed in an incubator at 30°C without shaking. Plasmids used in this study were maintained in the *Escherichia coli* DH5α-T1R strain (Invitrogen, Carlsbad, CA) and manipulated as previously described [90]. All microbial strains were handled under appropriate Biosafety Level 2 conditions (BSL2). All chemicals for media, buffer, and supplements were purchased from Difco Laboratories (Sparks, MD) or Sigma-Aldrich (Saint Louis, MO) unless otherwise stated.

Liquid YPD or YPD agar plates were supplemented with FK506 (1 µg/mL) or cyclosporine A (100 µg/mL). Various concentrations of FK506 were also used (0.025 to 1 µg/mL). FK506 and CsA were obtained from Astellas Pharma Inc. (North Brook, IL, USA) and LC Laboratories (Woburn, MA), respectively.

### Identification of calcineurin pathway components, annotation of the calcineurin catalytic A subunit genes in *M. circinelloides*, and phylogenetic analysis

Three available zygomycete genomes were examined, including *M. circinelloides* (http://genome.jgi-psf.org/Mucci1/Mucci1.home.html), *R. delemar* (http://www.broadinstitute.org/annotation/genome/rhizopus_oryzae/MultiHome.html), and *P. blakesleeanus* (http://genome.jgi.doe.gov/Phybl2/Phybl2.home.html). To assess the calcineurin components, including the calcineurin catalytic A and regulatory B subunits, FK506 binding protein 12 (FKBP12), cyclophilin A, calmodulin, and calmodulin-dependent protein kinase (CAM kinase), we applied a BLAST search by using *S. cerevisiae* and *C. neoformans* calcineurin pathway proteins. Proteins in the zygomycete genomes identified via a BLAST with *S. cerevisiae* and *C. neoformans* proteins were re-BLASTED against the *S. cerevisiae* and *C. neoformans* genomes. When the proteins in the zygomycete genomes returned the *S. cerevisiae* and *C. neoformans* counterpart proteins as the best hits, we defined them as the calcineurin components in the zygomycete genomes.

Fungal mass was lyophilized, and total RNA was extracted from the lyophilized fungal mass using the Ambion RiboPure-Yeast Kit (Invitrogen, Carlsbad, CA, USA). cDNA was synthesized from the extracted total RNA using the AffinityScript qPCR cDNA Synthesis Kit (Agilent Technologies, Santa Clara, CA, USA). The ORFs of the three *cna* genes were each amplified with a pair of primers: JOHE23748 and JOHE23750 for *cnaA*; JOHE23751 and JOHE23753 for *cnaB*; JOHE23754 and JOHE23756 for *cnaC*. Primers used in this study are listed in Table S1. PCR products obtained were directly sequenced with the primers above and additionally with JOHE20751 for *cnaA*, JOHE23765 and JOHE23766 for *cnaB*, and JOHE23767 and JOHE23768 for *cnaC*.

Deduced amino acid sequences of the *cna* genes were aligned with CLUSTALW, along with the calcineurin catalytic A subunit protein sequences from *R. delemar*, *P. blakesleeanus*, *S. cerevisiae*, *Aspergillus nidulans*, *C. neoformans*, and *Homo sapiens*. Phylogenies were constructed using a PhyML 3.0 software [91], which allowed phylogenies to be inferred and levels of support ascertained. Phylogenetic trees were drawn with the Dendroscope program [92] with aligned sequences.

### Generation of spontaneous mutants that are resistant to FK506 and sensitive to rapamycin

Calcineurin mutants MSL11 (*CNBR-1*), MSL12 (*CNBR-2*), MSL13 (*CNAA-1*), MSL14 (*CNAA-2*), MSL15 (*CNBR-1*), and MSL16 (*CNBR-3*) were isolated by placing a drop with *M. circinelloides* spores (R7B, MU406, MU407, MU420, MU416, and MU402 strains, respectively) (Table 1) in the center of YPD solid medium containing FK506 (1 µg/mL), as described before [65]. Between 5 days and 2 or 3 weeks of growth, some of the yeast colonies showed resistant mycelial outgrowths, from which spores were collected and passaged on FK506-containing solid medium until homokaryotic mycelial growth was observed. Spore suspensions were collected and tested for rapamycin resistance on YPD solid medium containing rapamycin (100 µg/mL). Total genomic DNA was isolated from the mutants and the genes encoding *fkbA*, *cnbR*, *cnaA*, *cnaB*, and *cnaC* were sequenced.

### GenBank accession numbers

Wild-type and mutant allele sequences obtained for *cnbR*, *cnaA*, *cnaB*, and *cnaC* were deposited in the GenBank under the following accession numbers, KC460400 (*cnbR*), KC460401 (*cnaA*), KC460402 (*cnaB*), KC460403 (*cnaC*), KC503248 (*CNBR-1*), KC503249 (*CNBR-2*), KC512815 (*CNBR-3*), KC503250 (*CNAA-1*), and KC503251 (*CNAA-2*).

### Disruption of *cnbR*, the calcineurin regulatory B subunit gene, and *cnaA*, a calcineurin catalytic A subunit gene

To disrupt the *cnbR* gene, we constructed a disruption allele containing the *pyrG* gene flanked by ∼1 kb of the 5′ and 3′ untranslated regions of the *cnbR* gene via overlap PCR. The 5′ region was amplified with primers JOHE22226 and JOHE22227, and the 3′ region was amplified with primers JOHE22230 and JOHE22231 from the genomic DNA of wild-type *M. circinelloides* strain CBS277.49 (the genome sequence reference strain). The *pyrG* gene was also amplified with primers JOHE22228 and JOHE22229 from CBS277.49 genomic DNA. The three fragments were then subjected to an overlap PCR with nested primers JOHE22236 and JOHE22237 to isolate a disruption allele as described [93]. The *cnbRΔ*::*pyrG* cassette was purified and cloned into plasmid pCR2.1-TOPO following the manufacturer's instructions (Invitrogen, Carlsbad, CA). The resulting plasmid, pCnbR-KO, was used to generate a greater amount of disruption construct DNA via PCR.

Strain MU402 (*leuA^−^*, *pyrG^−^*) was transformed with the obtained disruption cassette to disrupt the target *cnbR* gene, and transformation was carried out as described previously [94]. In brief, protoplasts (confirmed by lysis in water) were obtained from 2.5×10^8^ germinated spores of strain MU402 (*pyrG^−^*, *leuA^−^*) [95] by incubation with 0.03 unit/mL chitosanase RD (US Biological, Marblehead, MA) and 1 mg/mL lysing enzymes (L-1412; Sigma-Aldrich, Saint Louis, MO) at 30°C for 90 min. Protoplasts were incubated with 5 µg of DNA and electroporation was performed in 0.2 cm cuvettes (pulse at 0.8 kV, 25 µF, and 400 Ohms). *pyrG* ^+^ transformants were selected on MMC medium (1% casamino acids, 0.05% yeast nitrogen base without amino acids and ammonium sulfate, 2% glucose) pH 3.2, supplemented with 0.5 M sorbitol [95]. *Mucor* is coenocytic (aseptate), and therefore, an intensive selection procedure is required to obtain progeny with a homozygous karyotype. Thirty-nine transformants were obtained from eight independent transformations. The transformants were then grown in MMC selective medium for several vegetative cycles to increase the proportion of transformed nuclei. Spores of the transformants were spread onto MMC medium with uridine (200 mg/L) and MMC medium without uridine to determine the ratio between *pyrG* ^+^ and *pyrG* ^−^ progeny. Once the ratio reached 1∶1, genomic DNA from the transformants was extracted and used as a template for 5′ and 3′ junction PCRs to identify transformants in which homologous replacement of the *cnbR* locus with the *pyrG* allele had occurred. We found two independent transformants that contained the *cnbRΔ*::*pyrG* allele. Then, the two transformants underwent intensive vegetative selection cycles until all progeny were *pyrG* ^+^, and 5′ and 3′ junction PCRs then allowed us to confirm the disruption of the *cnbR* gene (Figure S8). JOHE22226 and JOHE37644 were used to amplify the 5′ junction and JOHE22231 and JOHE37645 to amplify the 3′ junction. Primers JOHE22226 and JOHE22231 are specific to the 5′- and 3′ regions outside of and upstream and downstream of the disruption cassette, respectively and were used for ORF spanning PCR. Primers JOHE37644 and JOHE37645 are specific to the marker gene *pyrG*. Primers For Southern blot analysis, primers JOHE22237 and JOHE39531 were used to amplify the probe (Figure S9).

Disruption of the *cnaA* gene was performed with the primers listed in Table S1. Thirty-seven transformants were obtained from eight independent transformations, and then screened for homologous recombination as described above. The *cnaA* gene disruption was confirmed by the same procedure described above. Primers JOHE26840 and JOHE37644 were used to amplify the 5′ junction and primers JOHE26845 and JOHE37645 to amplify the 3′ junction (Figure S11). Primers JOHE26840 and JOHE26845 were also used for ORF spanning PCR. The plasmid containing the *cnaA* disruption cassette in pCR2.1-TOPO was designated pAL1-1 (Table 1). Primers JOHE26847 and JOHE39530 were used to amplify the probe for Southern blot analysis (Figure S12).

### Virulence test

Wild-type and the *cnaAΔ* mutant spores were obtained from cultures on solid YPG media that were incubated for 4 days in the light. Sterile deionized water was then added to the media and spore suspensions were collected by scraping with a spreader. For the wild-type yeast, spores were inoculated at the bottom of a 500 mL flask completely filled with liquid YPD media and the flask was incubated without shaking to render the growth conditions anaerobic. The *cnbRΔ* mutant yeast were grown on YPD agar (pH = 6.5) for one day at 30°C and yeast colonies were scraped and resuspended in sterile deionized water. All inocula were washed twice with sterile PBS and quantified with a hemocytometer. Inocula in PBS were injected into wax moth larvae, and the survival rate of the host was monitored. The significance of the mortality rate data was evaluated by using Kaplan–Meier survival curves with the PRISM statistics software (GraphRad Software, Inc.).

### cAMP-dependent protein kinase A activity assay

Each strain of *Mucor*, *R. delemar* RA99-880, and *C. neoformans* H99 was grown in appropriate conditions. Crude protein extracts were obtained with radioimmunoprecipitation (RIPA) lysis buffer (Santa Cruz Biotechnology, Inc., Santa Cruz, CA) containing PMSF, sodium orthovanadate (a phosphatase inhibitor), and protease inhibitor cocktail solutions as recommended by the manufacturer. In brief, total fungal cells were briefly washed with PBS and soaked in the RIPA buffer with microbeads (426 to 600 µm, Sigma-Aldrich, Saint Louis, MO). Each sample was vigorously shaken in a bead beater 5 times for 1 minute each, rested for 5 minutes at 4°C, and centrifuged for 15 minutes at 12,000 rpm. Supernatants containing crude protein extracts were transferred into a new tube. Total protein was quantified with Bradford solution (BioRad, Hercules, CA) using a standard curve with bovine serum albumin. Protein kinase A activity of the crude protein extracts was measured with the SignaTECT cAMP-Dependent Protein Kinase (PKA) Assay System (Promega, Madison, WI) following the manufacturer's instructions. Three independent experiments were performed with consistent results.

### Real-time PCR

To determine the level of expression of the *cnaA*, *cnaB*, and *cnaC* genes during hyphal and yeast growth, quantitative RT-PCR assays were performed. Based on the ORF sequences, we designed pairs of specific RT-PCR primers across two exons of each *cna* gene: JOHE24019 and JOHE24020 for *cnaA*; JOHE24021 and JOHE24022 for *cnaB*; JOHE24023 and JOHE24024 for *cnaC* and JOHE24075 and JOHE24076 for actin. qRT-PCR was performed using the Brilliant SYBR Green QPCR Master Mix (Agilent Technologies), and the expression of the actin gene was used for normalization. The expression levels of the *cna* genes were evaluated by ct values from RT-PCR. Multiple independent experiments were performed with concordant results.

### Microscopy

Spores and hyphae were observed with a Zeiss Axioskop 2 Plus equipped with an AxioCam MRm camera (Carl Zeiss Inc., Thornwood, NY). To analyze nuclei, spores were fixed with 3.7% formaldehyde in 50 mM potassium phosphate buffer, pH 7.0, containing 0.2% Triton X-100 (v/v). The specimens were then mounted on a glass slide with ProLong Gold antifade reagent with DAPI (Invitrogen, Carlsbad, CA).

Time lapse analyses for the germination of wild-type and *cnaA* mutants were performed by using a Zeiss Axio Observer Z1 microscope system (Carl Zeiss Inc., Thornwood, NY) with an Opto-electronically motorized XY stage, Pecon XL S1 incubator, and Coolsnap ES2 high-resolution CCD camera, which are housed in the Duke University Light Microscopy Core Facility (LMCF). The images were sequentially obtained every 30 seconds and combined as a movie (30 frames per second) by using MetaMorph 7.6.5 (Molecular devices Inc., Sunnyvale, CA).

## Supporting Information

Figure S1 Phenotypes of *M. circinelloides* grown in the presence of calcineurin inhibitors FK506 or cyclosporine A (CsA). FK506 induces yeast growth; however, exposure to the other calcineurin inhibitor CsA resulted in abnormal hyphal growth instead of inducing yeast growth. FK506-specific inhibition of calcineurin may drive *Mucor* to grow as yeast. Alternatively, CsA may not be fully functional in this organism, for example, CsA less efficiently inhibits calcineurin (See text for detailed discussion). Scale = 10 µm.
(TIF)

Figure S2 Yeast growth of *Mucor* induced by a combination of cyclosporine A and FK506. CsA (100 mg/L) alone does not fully induce yeast growth. At a sub-active concentration (0.3 µg/L), FK506 (0.3×) can partially inhibit hyphal growth without inducing yeast growth. When CsA was combined with sub-active FK506, however, *Mucor* fully exhibited yeast growth. This result indicates that CsA may not be as active as FK506 in *Mucor*. Scale = 20 µm.
(TIF)

Figure S3 Conserved calcineurin components in three zygomycetes and evolutionary trajectory of the catalytic A subunit in the Mucorales fungi. (A) High numbers of calmodulin and calmodulin (CAM) kinase orthologs were identified in the three zygomycete genomes. *M. circinelloides* and *R. delemar* each have nine calmodulins while *P. blakesleeanus* has six, and *M. circinelloides* and *P. blakesleeanus* each have six CAM kinases while *R. delemar* has eight. Interestingly, two pathogenic zygomycetes, *M. circinelloides* and *R. delemar*, each encode three calcineurin catalytic A subunits, whereas the non-pathogenic species *P. blakesleeanus* only encodes one. *M. circinelloides* and *P. blakesleeanus* have a single calcineurin regulatory B subunit and cyclophilin A gene, whereas *R. delemar* encodes two paralogs of each gene, which may be the result of a whole genome duplication event unique to this lineage. All three species have one FKBP12 gene. (B) Phylogenetic analyses revealed that a common branch, including McCnaA, PbCnaA, RdCnaA, and RdCnaB, is conserved in the three zygomycete species. This result indicates that the calcineurin A subunit gene in this group may be the ancestral one and a duplication event might have generated additional *cna* genes in the *Mucor* and *Rhizopus* lineages (solid red arrow). Another independent duplication may have produced the third *cna* gene in the *Mucor* lineage (open red arrow). The RdCnaA and RdCnaB duplicated subunits in the *Rhizopus* lineage likely resulted from a recent whole genome duplication event; this hypothesis is supported by the short branch length and conserved flanking genes around the *cna* genes. Hs: *Homo sapiens*, Sc: *S. cerevisiae*, Cn: *C. neoformans*, An: *A. nidulans*, Mc: *M. circinelloides*, Rd: *R. delemar*, and Pb: *Phycomyces blakesleeanus*.
(TIF)

Figure S4 Amino acid sequence comparisons between CnbR-1, CnbR-2, CnbR-3 and CnbR. The *CNBR-1* allele encodes tyrosine (Y) instead of asparagine (N) in *cnbR* at the 125^th^ residue; the *CNBR-2* allele encodes an additional histidine (H) inserted at the 130^th^ residue; and the *CNBR-3* allele encodes phenylalanine (F) instead of valine (V) in *cnbR* at the 122^nd^ residue. The amino acid alterations occurred in the latch region that interacts with FKBP12-FK506 and is also involved in the phosphatase activity of calcineurin. This modification may result in the resistance of the mutants to FK506.
(TIF)

Figure S5 An N125Y substitution in the calcineurin regulatory B subunit results in resistance to FK506 but hypersensitivity to CsA. (A) The *CNBR-1* (N125Y) mutant is resistant to FK506, forming hyphal growth in the presence of FK506 (1 µg/L), and under this condition the wild-type only grows as yeast. However, the *CNBR-1* mutant displaysed higher sensitivity to CsA (100 mg/L) compared to the other *CNBR* mutants: the *CNBR-1* mutant grew much slower than the *CNBR-2* and *fkbAΔ* mutants. The *fkbAΔ* mutant was also resistant to rapamycin, whereas *CNBR-1* and *CNBR-2* mutants were sensitive. (B) When observed under the microscope, the two *CNBR-1* mutants exhibited yeast growth in the presence of CsA (100 mg/L), whereas the wild-type displayed abnormal hyphal growth. Scale = 20 µm.
(TIF)

Figure S6 A V122F substitution in the calcineurin regulatory B subunit confers cross-resistance to FK506 and CsA. The *CNBR-3* (V122F) mutant is resistant to both of FK506 and CsA, whereas the other calcineurin mutants, *CNBR-1*, *fkbAΔ*, and SM4 (L91P) mutants are resistant to FK506 but sensitive to CsA. The *fkbAΔ* mutant is resistant to rapamycin, whereas the SM4, *CNBR-1*, and *CNBR-3* mutants are sensitive.
(TIF)

Figure S7 Amino acid sequence comparisons between CnaA-1, CnaA-2, and CnaA. The *CNAA-1* allele encodes threonine (T) instead of serine (S) in *cnaA* at the 378^th^ residue; the *CNAA-2* allele encodes aspartic acid (D) instead of asparagine (N) in *cnaA* at the 370^th^ residue. The amino acid alterations are present in the binding domains for calcineurin B and for the FKBP12-FK506 complex and, therefore, the interaction between FKBP12-FK506 and calcineurin may be modified to confer resistance to FK506.
(TIF)

Figure S8 PCR confirmation of the disruption of the *cnbR* gene. (A) Illustration of the *cnbRΔ*::*pyrG* and *cnbR* alleles with ∼1 kb of up- and downstream sequence. P1 and P4 primers recognize sequences outside of the disruption cassette. P2 and P3 primers recognize the *pyrG* gene. P1, JOHE22226; P2, JOHE37644; P3, JOHE37645; P4, JOHE22231 (Table S1). (B) P1 and P4 primers amplified a 4332 bp region of the *cnbRΔ*::*pyrG* allele from two independent *cnbR* mutants, whereas the same primers amplified only a 2958 bp *cnbR* fragment from wild-type. P1 and P2 primers amplified 1658 bp from the 5′ junction of the *cnbRΔ*::*pyrG* allele, and the P3 and P4 primers amplified 1895 bp from the 3′ junction of the *cnbRΔ*::*pyrG* allele. The same pairs of primers did not produce junction fragments from the wild-type. The gene and primer sizes are not to scale. M1: MSL7 and M2: MSL8 (Table 1).
(TIF)

Figure S9 Southern blot confirmation of the disruption of the *cnbR* gene. Genomic DNA (30 µg) of wild-type (MU402), MSL7 (*cnbRΔ1*), and MSL8 (*cnbRΔ2*) were fully digested with BamHI. The 3′ UTR end of the *cnbR* gene was amplified and labeled with P^32^. The probe sequence is a part of the disruption cassette. The probe detected a 13,105 bp wild-type BamHI fragment, whereas a 4,650 bp BamHI fragment was detected in the *cnbRΔ* mutants. This confirms a deletion of the *cnbR* gene in the *cnbRΔ* mutants. No apparent extra signals were detected, indicating that no ectopic integration events occurred. The gene sizes are not to scale.
(TIF)

Figure S10 Morphology of *M. circinelloides* in infected host. (A) Wax moth larvae were infected with *Mucor* (20,000 spores or yeast) and after two days, the larvae were frozen and sliced for microscopic observation. Fungal mass in tissues was stained with a 0.05% solution of calcofluor white. The wild-type displayed hyphal growth inside of infected tissues, whereas the two independent *cnbRΔ* mutants only grew as yeast inside of the wax moth larva hosts. Scale = 20 µm. CFW: calcofluor white, DIC: differential interference contrast. (B) Mice were infected with *Mucor* or *Rhizopus*, and the brains from infected mice were collected and tissue specimens were stained with Gomori's methenamine silver (GSM) at 2 days post infection. Both fungi displayed hyphal growth in the recovered brain tissues. Scale = 50 µm.
(TIF)

Figure S11 FK506 increased cAMP-dependent protein kinase A (PKA) activity in two other pathogenic fungi, *R. delemar* and *C. neoformans*. (A) When treated with FK506, PKA activity was elevated in *R. delemar*. (B) Calcineurin is known to function at 37°C in the basidiomycete pathogen *C. neoformans*. At this host temperature, FK506 treatment resulted in higher PKA activity.
(TIF)

Figure S12 PCR confirmation of *cnaA* gene disruption. (A) Illustration of the *cnaAΔ*::*pyrG* and *cnbA* alleles with ∼1 kb of up- and downstream sequence. P1 and P4 primers recognize sequences outside of the disruption cassette. P2 and P3 primers recognize the *pyrG* gene. P1, JOHE26840; P2, JOHE37644; P3, JOHE37645; P4, JOHE26845 (Table S1). (B) P1 and P4 primers amplified a 4447 bp product for the *cnaAΔ*::*pyrG* allele from two independent *cnaA* mutants, whereas the same primers amplified only a 4791 bp *cnaA* fragment from wild-type. P1 and P2 primer amplified a 1582 bp region from the 5′ junction of the *cnaAΔ*::*pyrG* allele, and the P3 and P4 primers a 2113 bp product from the 3′ junction of the *cnaAΔ*::*pyrG* allele from the two *cnaA* mutants. The same pairs of primers did not produce junction fragments from wild-type. The gene and primer sizes are not to scale. M1: MSL9 and M2: MSL10 (Table 1).
(TIF)

Figure S13 Southern blot confirmation of the disruption of the *cnaA* gene. Genomic DNA (30 µg) of wild-type (MU402), MSL9 (*cnaAΔ1*), and MSL10 (*cnaAΔ2*) were fully digested with BamHI. The 3′ UTR end of the *cnaA* gene was amplified and labeled with P^32^. The probe sequence is a part of the disruption cassette. The probe detected a 10,461 bp wild-type BamHI fragment, whereas a 5,442 bp BamHI fragment was detected in the *cnaAΔ* mutants, confirming a deletion of the *cnaA* gene in the *cnaAΔ* mutants. No apparent extra signals were detected, indicating that no ectopic integration events occurred. The gene sizes are not to scale.
(TIF)

Figure S14 Morphology of *cnaA* mutants in the wax moth larva host. Wild-type and two independent *cnaAΔ* mutants (20,000 spores) were inoculated into wax moth larvae. After two days, the larvae were frozen and sliced for microscopic observation. A drop of 0.05% calcofluor white solution was applied to stain fungal mass in the tissues. Both of the wild-type and *cnaAΔ* mutants formed hyphae inside of the wax moth larva hosts. Scale = 10 µm.
(TIF)

Figure S15 Larger spores of the *cnaA* mutants germinate earlier than wild-type. J774.A1 macrophage cells (5×10^5^) were inoculated into each well of a six-well plate. After 24 hours of incubation, 5×10^5^ spores were co-cultured with the macrophages. The interactions between macrophages and spores were observed every 30 minutes for 6 hours. The larger *cnaA* spores germinated earlier, at 3.5 hours, inside macrophages than the wild-type, at 6 hours. The images were taken at 3.5 hour.
(TIF)

Figure S16 Effect of FK506 on diabetic (upper) and non-diabetic (bottom) murine hosts infected with *M. circinelloides* or *R. oryzae*. Groups of BALB/c mice were rendered diabetic with streptozocin (190 mg per body kg) through intraperitoneal injection 10 days prior to fungal challenge [53], [96]. The mice were infected with 10^4^ *Mucor* (CNRMA04.805) spores or 10^3^ *Rhizopus* (RA99-880) spores in 200 µL PBS via tail vein injection. The survival rate of the hosts was monitored twice a day, and body weight was measured daily. Animals that appeared moribund or in pain were sacrificed. The significance of mortality data was evaluated with Kaplan-Meier survival curves. The mice infected with *Mucor* or *Rhizopus* spores were immediately administered FK506 at a concentration of 5 mg per kg of body weight via intraperitoneal injection. Two additional FK506 treatments were performed at the same concentration at 24 and 48 hours post-infection. In both groups of mice infected with *Mucor* or *Rhizopus*, no apparent therapeutic effect of FK506 was observed. Non-diabetic murine host models were also tested, in which 10^5^ *Mucor* spores and 10^4^ *Rhizopus* spores were intravenously injected. FK506 at 5 mg per kg of body weight was administered at 0, 24, and 48 hours post-infection. No apparent therapeutic benefit of FK506 was observed in this analysis. A detrimental effect was observed in the mice infected with *Mucor* (*p* = 0.0015). It is possible that in both the diabetic and non-diabetic murine host models immunosuppression due to FK506 might have counteracted the antifungal effects of the drug. These results suggest that lower doses of FK506 (altered dosage regimens), less or non-immunosuppressive FK506 analogs, or a combination of FK506 with other antifungal drugs will be necessary for therapeutic efficacy.
(TIF)

Figure S17 One hundred kilobases upstream and downstream of the three *cna* genes are depicted. The 5′ and 3′ flanking regions of the three *cna* genes are not syntenic; however, repetitive elements are present. The blue-arrow repetitive sequences (∼1 kb) are found in the flanking areas of all three *cna* genes; the green-arrow repetitive elements (∼500 bp) are found in the flanking areas of the *cnaA* and *cnaC* genes; the red-arrow and gray-arrow repetitive elements are found only in the flanking area of the *cnaA* or *cnaB* gene, respectively. These observations illustrate that the three *cna* genes may have evolved from multiple segmental gene duplication events, which may have been facilitated by these or other repetitive elements.
(TIF)

Table S1 Primers used in this study.
(DOC)

Video S1 Hyphal growth of *M. circinelloides*. The wild-type R7B strain was grown on YDP solid media in aeration conditions. *Mucor* exhibited a filamentous growth forming mycelial complex. Speed = 900×.
(MOV)

Video S2 Yeast growth of M. circinelloides. The R7B strain grows solely as multi-budded yeast in the presence of FK506 (1 µg/mL). Speed = 900×.
(MOV)

Video S3 Yeast growth of *cnbR* mutant. The MSL7 (*cnbRΔ1*) strain grows solely as multi-budded yeast on YPD media. Speed = 900×.
(MOV)

Video S4 Hyphal growth of wild-type *M. circinelloides*. Wild-type hyphae elongates in a single direction. Speed = 900×.
(MOV)

Video S5 Hyphal growth of *cnaA* mutant. The MSL9 (*cnaAΔ1*) strain exhibits a tip-splitting phenotype and abnormal branching during hyphal growth. Speed = 900×.
(MOV)
