# Supplementary figures and images for: Calcineurin Plays Key Roles in the Dimorphic Transition and Virulence of the Human Pathogenic Zygomycete Mucor circinelloides
Source: PLoS Pathog. 2013 Sep 5;9(9):e1003625. doi: 10.1371/journal.ppat.1003625 (PMC3764228; doi:10.1371/journal.ppat.1003625)

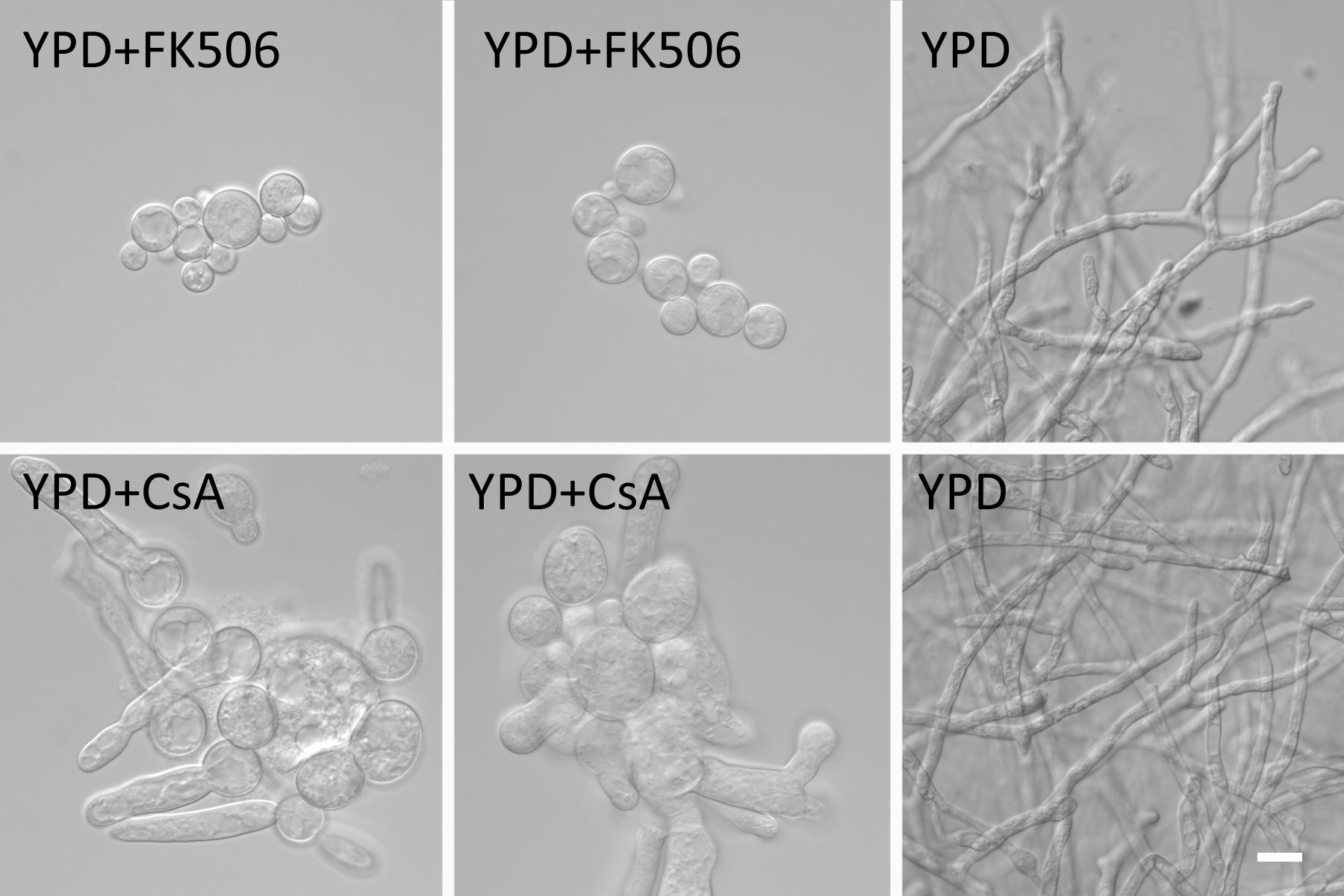

Supplement: Figure S1 — Phenotypes of M. circinelloides grown in the presence of calcineurin inhibitors FK506 or cyclosporine A (CsA). FK506 induces yeast growth; however, exposure to the other calcineurin inhibitor CsA resulted in abnormal hyphal growth instead of inducing yeast growth. FK506-specific inhibition of calcineurin may drive Mucor to grow as yeast. Alternatively, CsA may not be fully functional in this organism, for example, CsA less efficiently inhibits calcineurin (See text for detailed discussion). Scale = 10 µm. (TIF) [file ppat.1003625.s001.tif]

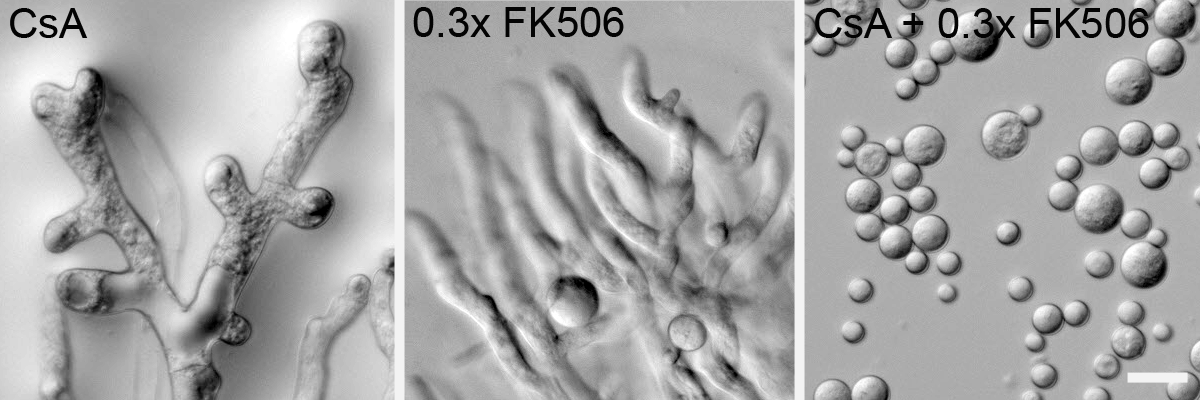

Supplement: Figure S2 — Yeast growth of Mucor induced by a combination of cyclosporine A and FK506. CsA (100 mg/L) alone does not fully induce yeast growth. At a sub-active concentration (0.3 µg/L), FK506 (0.3×) can partially inhibit hyphal growth without inducing yeast growth. When CsA was combined with sub-active FK506, however, Mucor fully exhibited yeast growth. This result indicates that CsA may not be as active as FK506 in Mucor. Scale = 20 µm. (TIF) [file ppat.1003625.s002.tif]

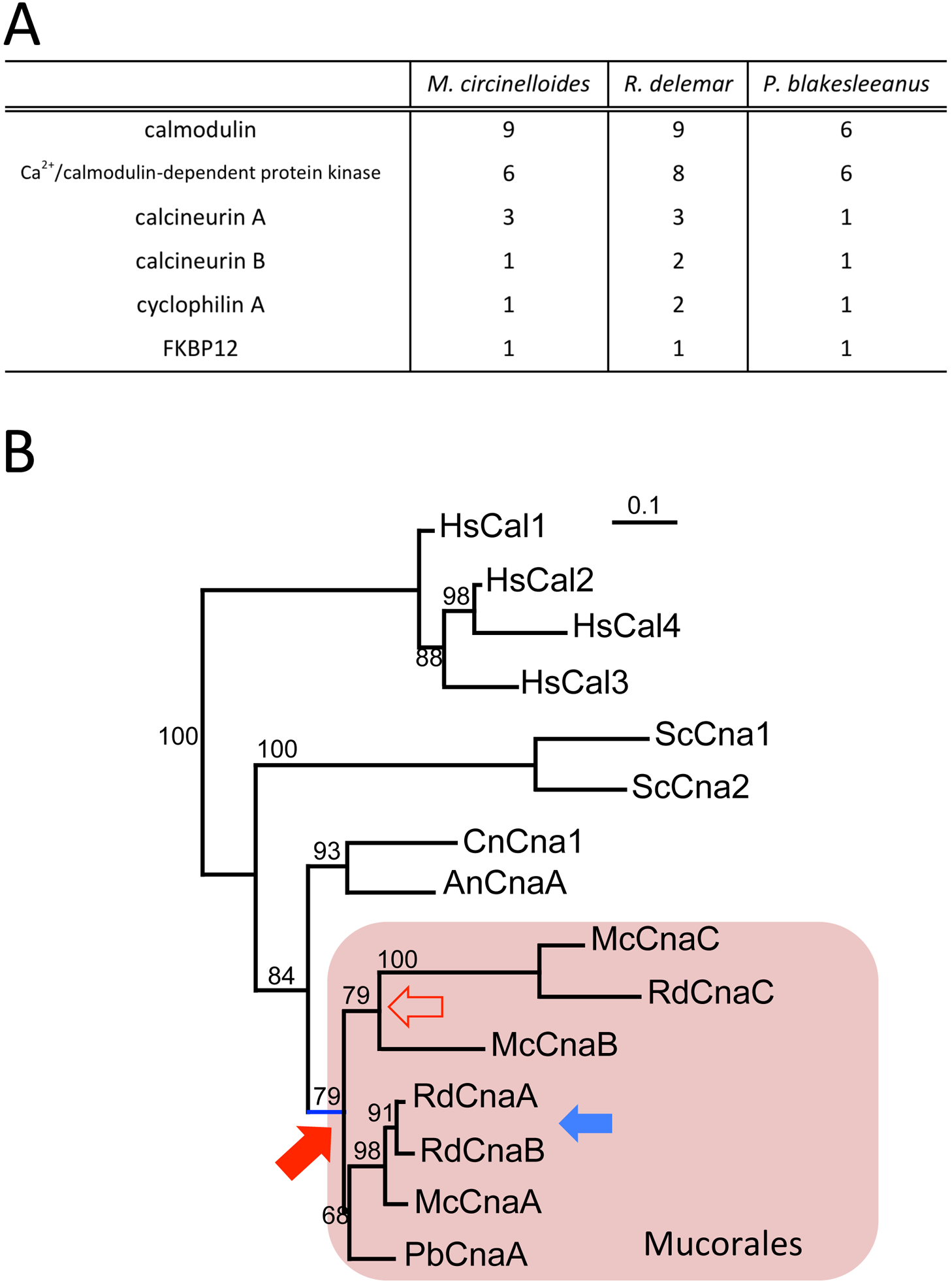

Supplement: Figure S3 — Conserved calcineurin components in three zygomycetes and evolutionary trajectory of the catalytic A subunit in the Mucorales fungi. (A) High numbers of calmodulin and calmodulin (CAM) kinase orthologs were identified in the three zygomycete genomes. M. circinelloides and R. delemar each have nine calmodulins while P. blakesleeanus has six, and M. circinelloides and P. blakesleeanus each have six CAM kinases while R. delemar has eight. Interestingly, two pathogenic zygomycetes, M. circinelloides and R. delemar, each encode three calcineurin catalytic A subunits, whereas the non-pathogenic species P. blakesleeanus only encodes one. M. circinelloides and P. blakesleeanus have a single calcineurin regulatory B subunit and cyclophilin A gene, whereas R. delemar encodes two paralogs of each gene, which may be the result of a whole genome duplication event unique to this lineage. All three species have one FKBP12 gene. (B) Phylogenetic analyses revealed that a common branch, including McCnaA, PbCnaA, RdCnaA, and RdCnaB, is conserved in the three zygomycete species. This result indicates that the calcineurin A subunit gene in this group may be the ancestral one and a duplication event might have generated additional cna genes in the Mucor and Rhizopus lineages (solid red arrow). Another independent duplication may have produced the third cna gene in the Mucor lineage (open red arrow). The RdCnaA and RdCnaB duplicated subunits in the Rhizopus lineage likely resulted from a recent whole genome duplication event; this hypothesis is supported by the short branch length and conserved flanking genes around the cna genes. Hs: Homo sapiens, Sc: S. cerevisiae, Cn: C. neoformans, An: A. nidulans, Mc: M. circinelloides, Rd: R. delemar, and Pb: Phycomyces blakesleeanus. (TIF) [file ppat.1003625.s003.tif]

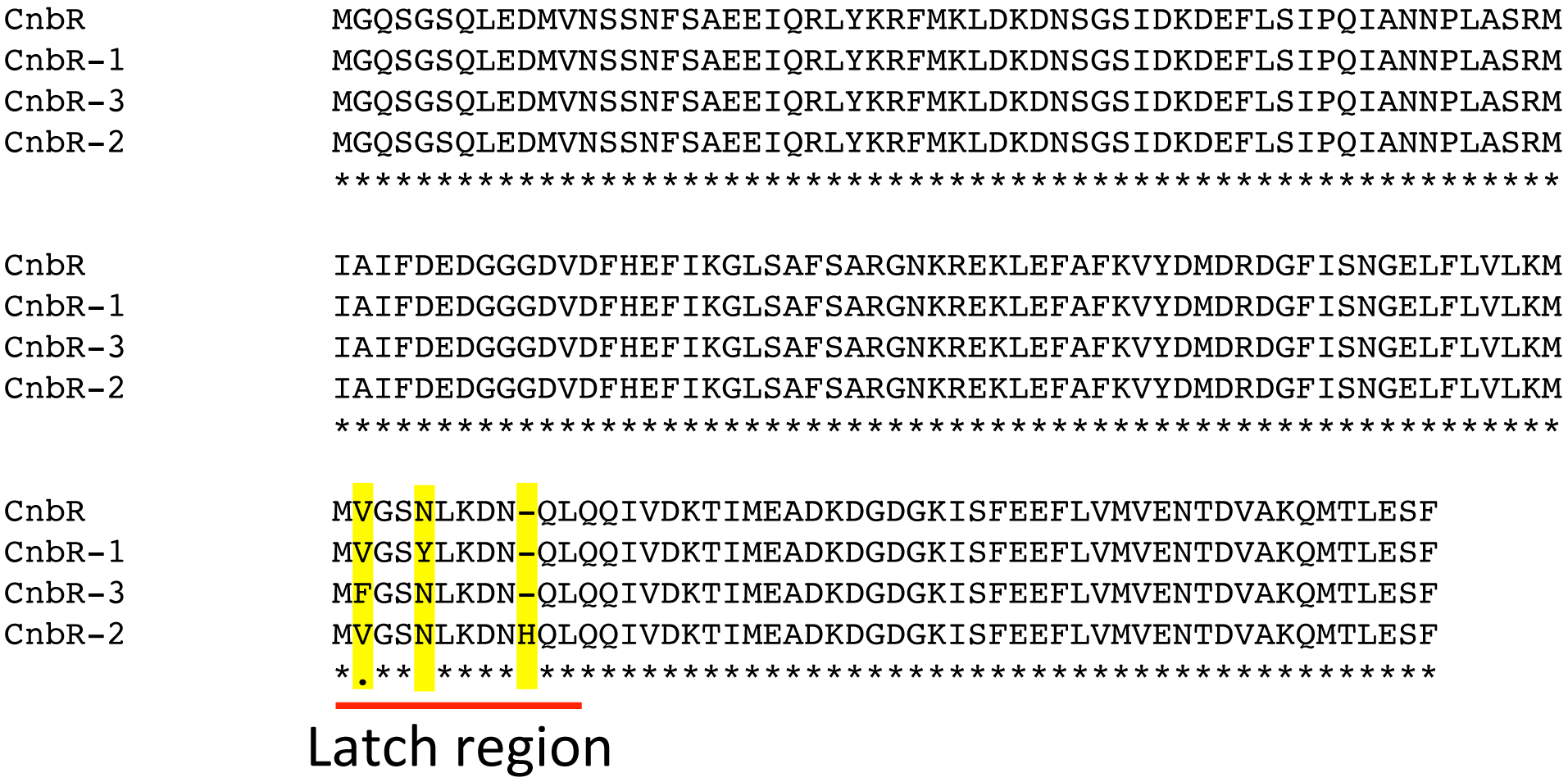

Supplement: Figure S4 — Amino acid sequence comparisons between CnbR-1, CnbR-2, CnbR-3 and CnbR. The CNBR-1 allele encodes tyrosine (Y) instead of asparagine (N) in cnbR at the 125th residue; the CNBR-2 allele encodes an additional histidine (H) inserted at the 130th residue; and the CNBR-3 allele encodes phenylalanine (F) instead of valine (V) in cnbR at the 122nd residue. The amino acid alterations occurred in the latch region that interacts with FKBP12-FK506 and is also involved in the phosphatase activity of calcineurin. This modification may result in the resistance of the mutants to FK506. (TIF) [file ppat.1003625.s004.tif]

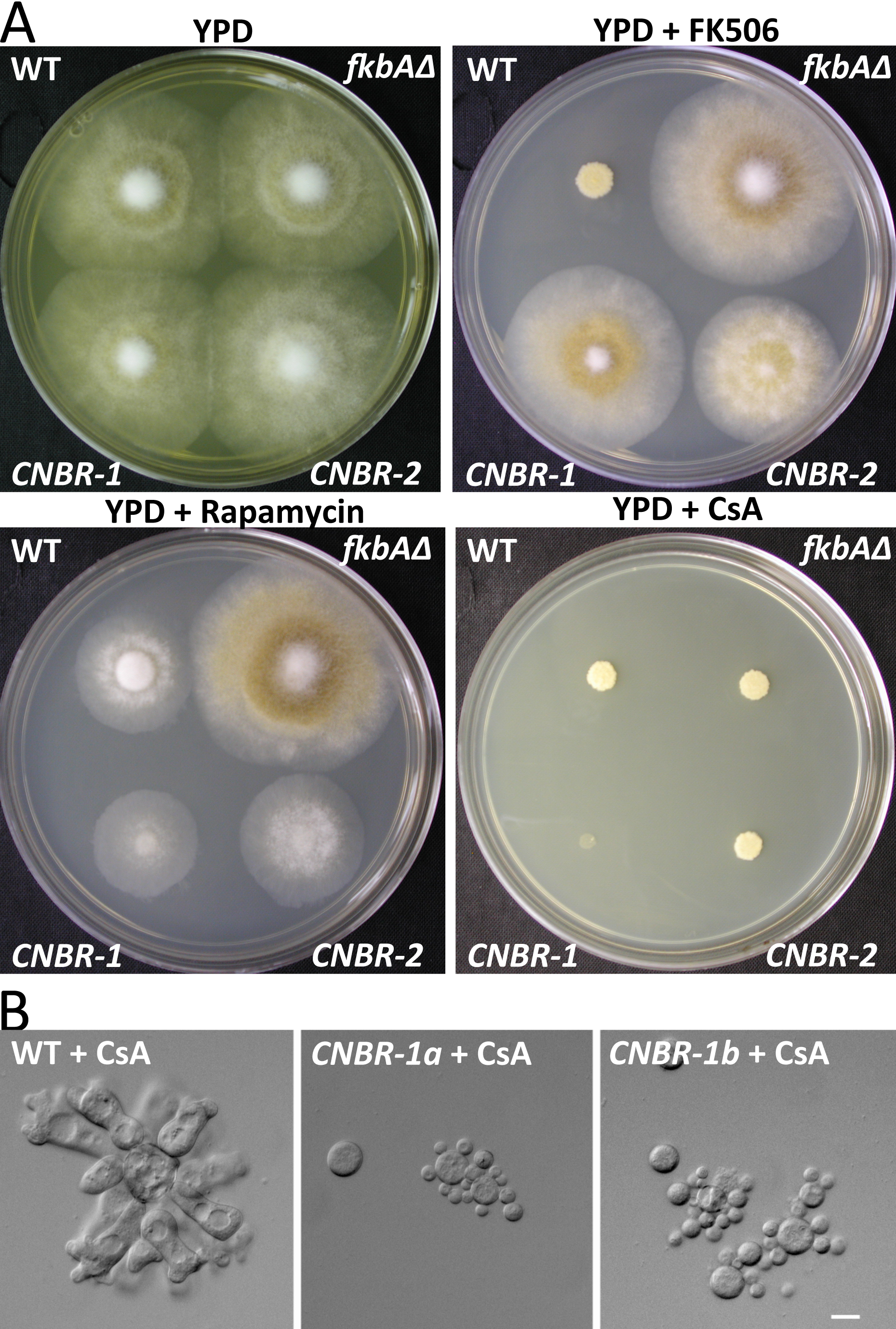

Supplement: Figure S5 — An N125Y substitution in the calcineurin regulatory B subunit results in resistance to FK506 but hypersensitivity to CsA. (A) The CNBR-1 (N125Y) mutant is resistant to FK506, forming hyphal growth in the presence of FK506 (1 µg/L), and under this condition the wild-type only grows as yeast. However, the CNBR-1 mutant displaysed higher sensitivity to CsA (100 mg/L) compared to the other CNBR mutants: the CNBR-1 mutant grew much slower than the CNBR-2 and fkbAΔ mutants. The fkbAΔ mutant was also resistant to rapamycin, whereas CNBR-1 and CNBR-2 mutants were sensitive. (B) When observed under the microscope, the two CNBR-1 mutants exhibited yeast growth in the presence of CsA (100 mg/L), whereas the wild-type displayed abnormal hyphal growth. Scale = 20 µm. (TIF) [file ppat.1003625.s005.tif]

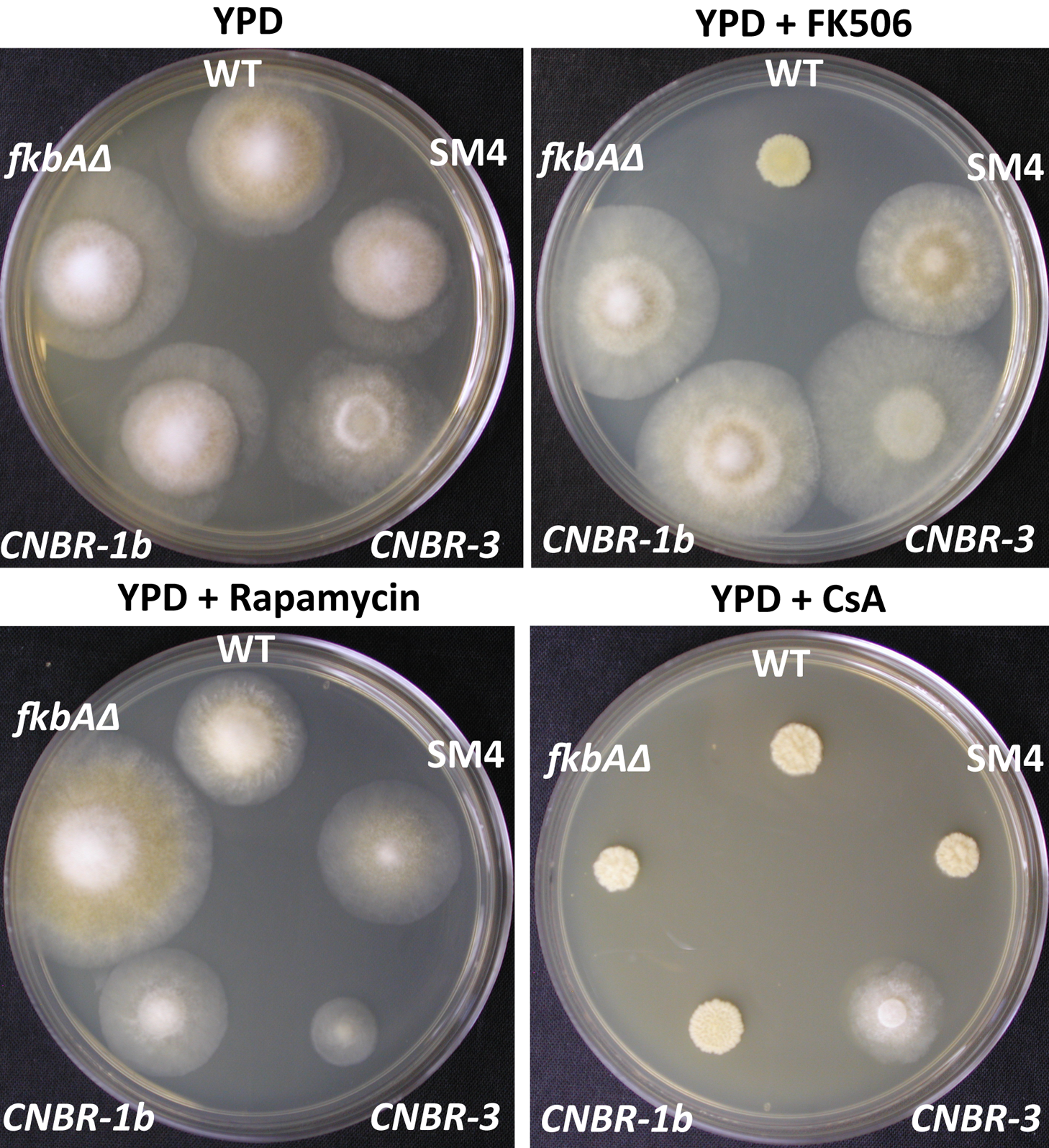

Supplement: Figure S6 — A V122F substitution in the calcineurin regulatory B subunit confers cross-resistance to FK506 and CsA. The CNBR-3 (V122F) mutant is resistant to both of FK506 and CsA, whereas the other calcineurin mutants, CNBR-1, fkbAΔ, and SM4 (L91P) mutants are resistant to FK506 but sensitive to CsA. The fkbAΔ mutant is resistant to rapamycin, whereas the SM4, CNBR-1, and CNBR-3 mutants are sensitive. (TIF) [file ppat.1003625.s006.tif]

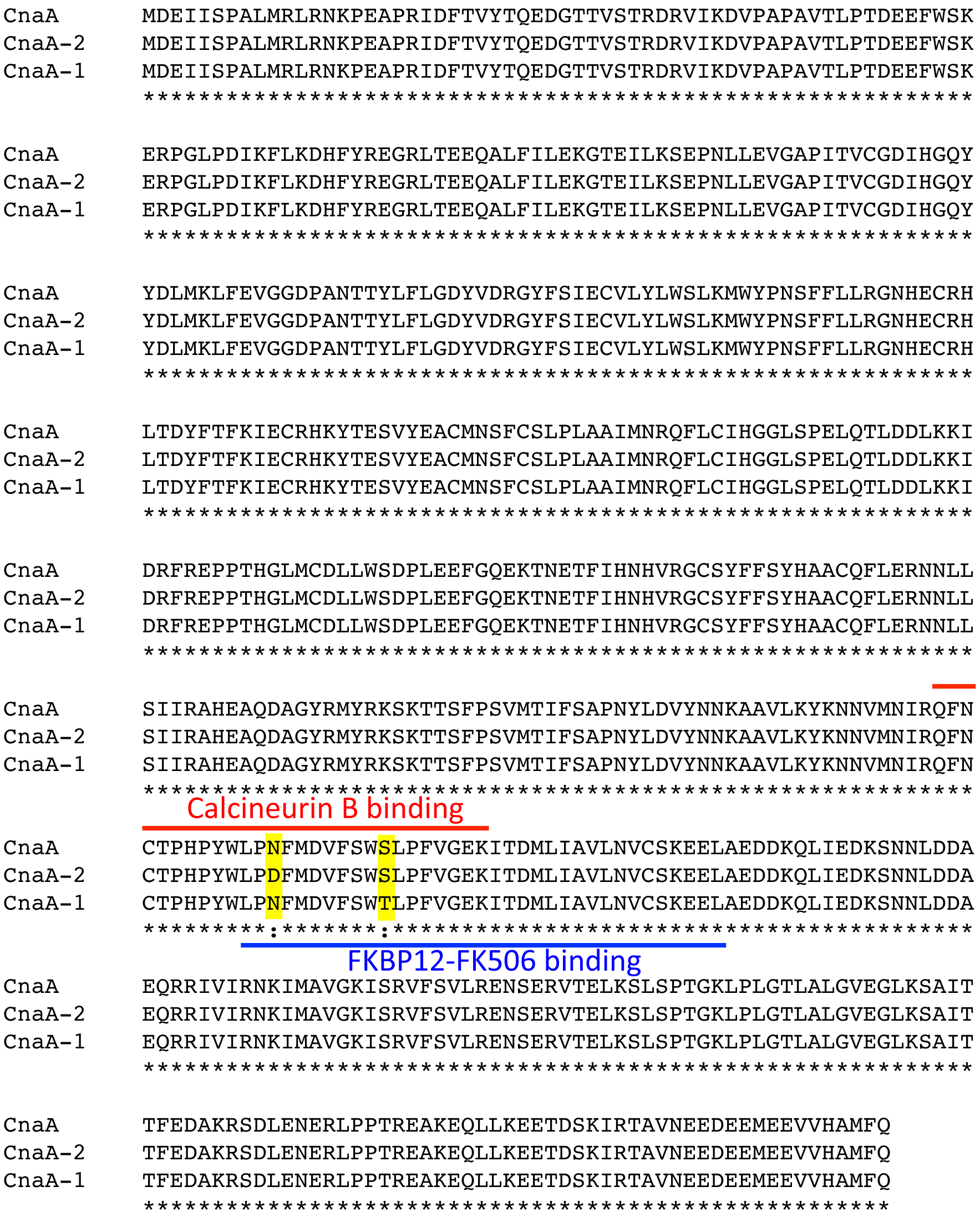

Supplement: Figure S7 — Amino acid sequence comparisons between CnaA-1, CnaA-2, and CnaA. The CNAA-1 allele encodes threonine (T) instead of serine (S) in cnaA at the 378th residue; the CNAA-2 allele encodes aspartic acid (D) instead of asparagine (N) in cnaA at the 370th residue. The amino acid alterations are present in the binding domains for calcineurin B and for the FKBP12-FK506 complex and, therefore, the interaction between FKBP12-FK506 and calcineurin may be modified to confer resistance to FK506. (TIF) [file ppat.1003625.s007.tif]

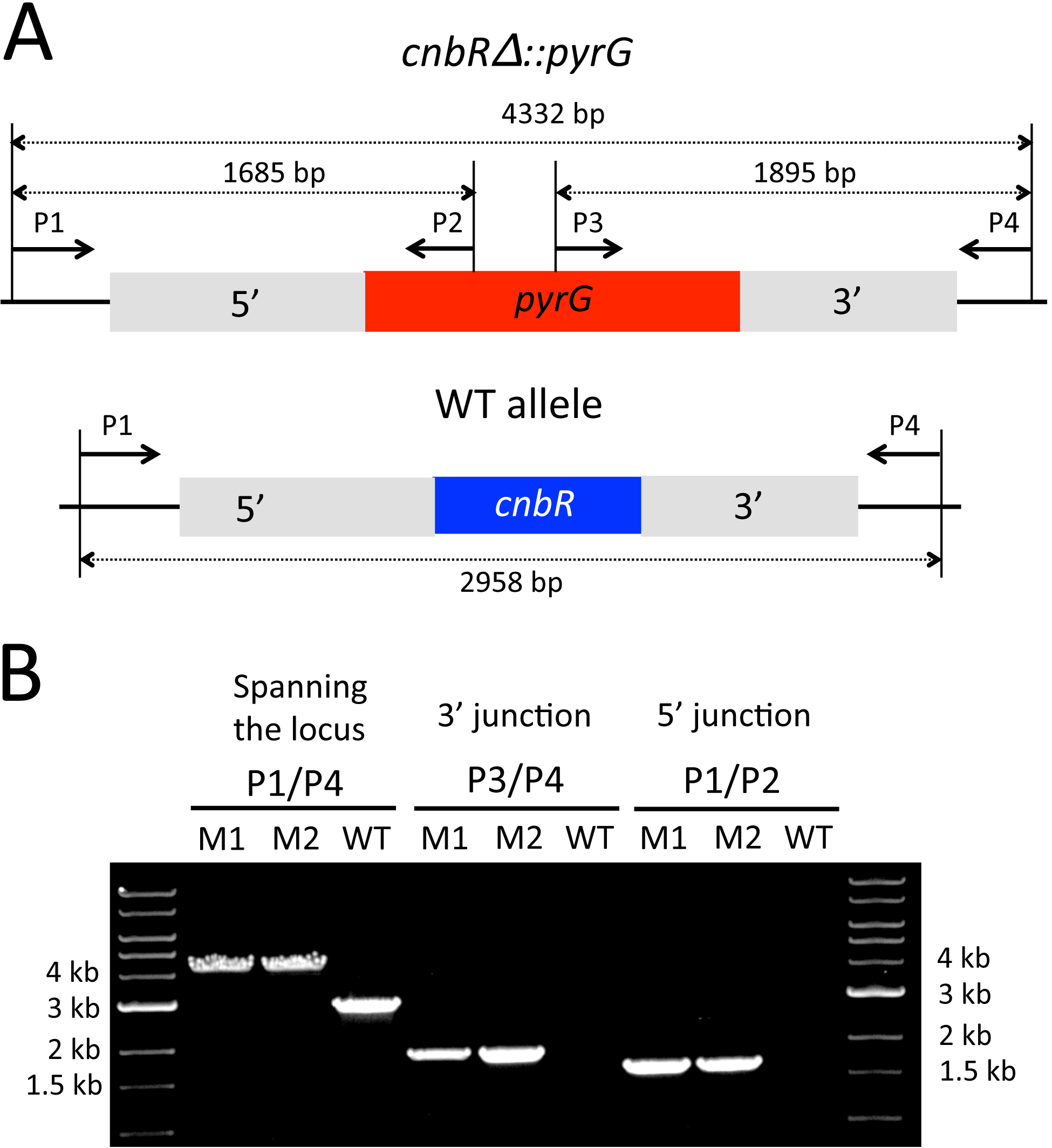

Supplement: Figure S8 — PCR confirmation of the disruption of the cnbR gene. (A) Illustration of the cnbRΔ::pyrG and cnbR alleles with ∼1 kb of up- and downstream sequence. P1 and P4 primers recognize sequences outside of the disruption cassette. P2 and P3 primers recognize the pyrG gene. P1, JOHE22226; P2, JOHE37644; P3, JOHE37645; P4, JOHE22231 (Table S1). (B) P1 and P4 primers amplified a 4332 bp region of the cnbRΔ::pyrG allele from two independent cnbR mutants, whereas the same primers amplified only a 2958 bp cnbR fragment from wild-type. P1 and P2 primers amplified 1658 bp from the 5′ junction of the cnbRΔ::pyrG allele, and the P3 and P4 primers amplified 1895 bp from the 3′ junction of the cnbRΔ::pyrG allele. The same pairs of primers did not produce junction fragments from the wild-type. The gene and primer sizes are not to scale. M1: MSL7 and M2: MSL8 (Table 1). (TIF) [file ppat.1003625.s008.tif]

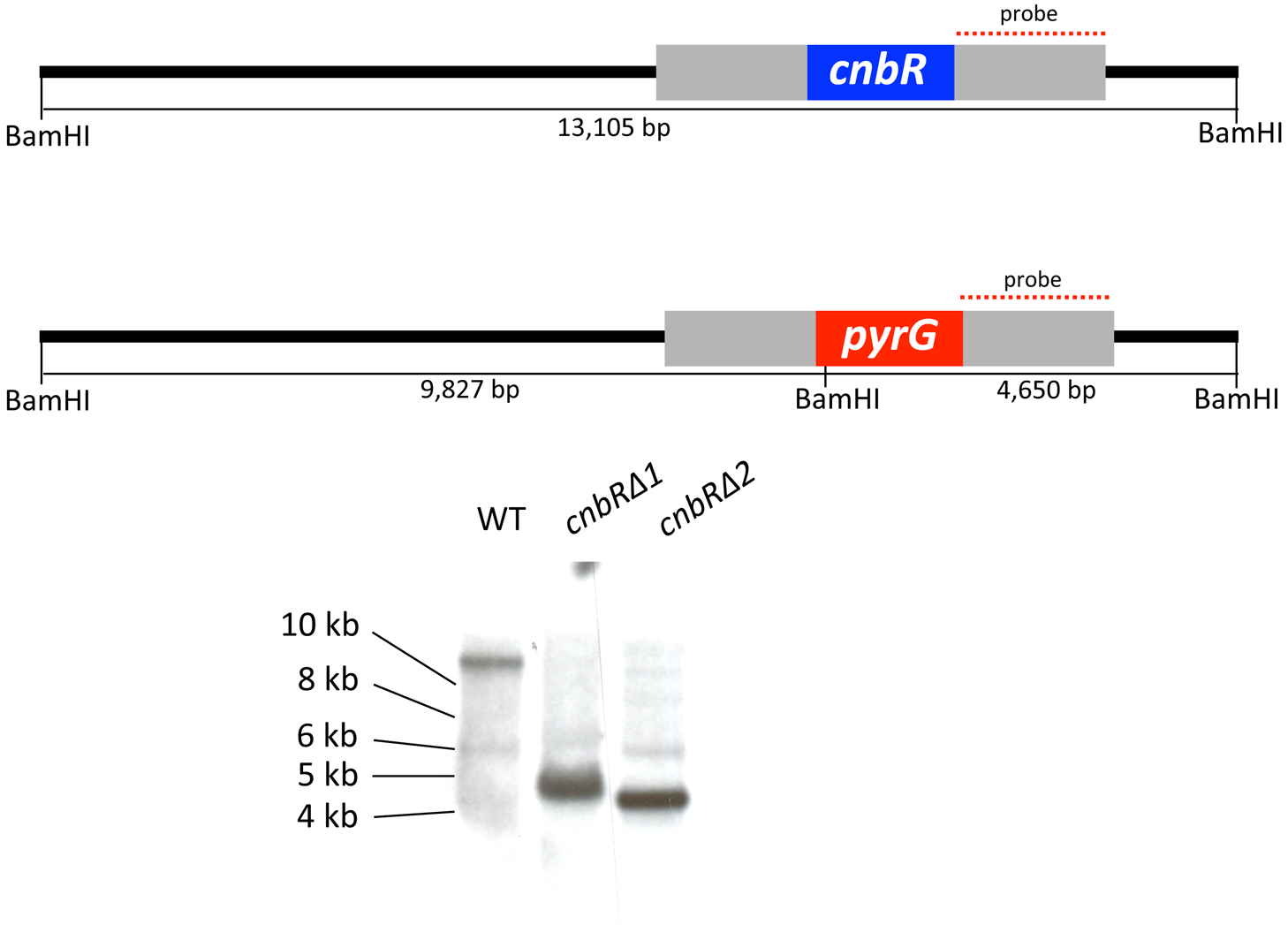

Supplement: Figure S9 — Southern blot confirmation of the disruption of the cnbR gene. Genomic DNA (30 µg) of wild-type (MU402), MSL7 (cnbRΔ1), and MSL8 (cnbRΔ2) were fully digested with BamHI. The 3′ UTR end of the cnbR gene was amplified and labeled with P32. The probe sequence is a part of the disruption cassette. The probe detected a 13,105 bp wild-type BamHI fragment, whereas a 4,650 bp BamHI fragment was detected in the cnbRΔ mutants. This confirms a deletion of the cnbR gene in the cnbRΔ mutants. No apparent extra signals were detected, indicating that no ectopic integration events occurred. The gene sizes are not to scale. (TIF) [file ppat.1003625.s009.tif]

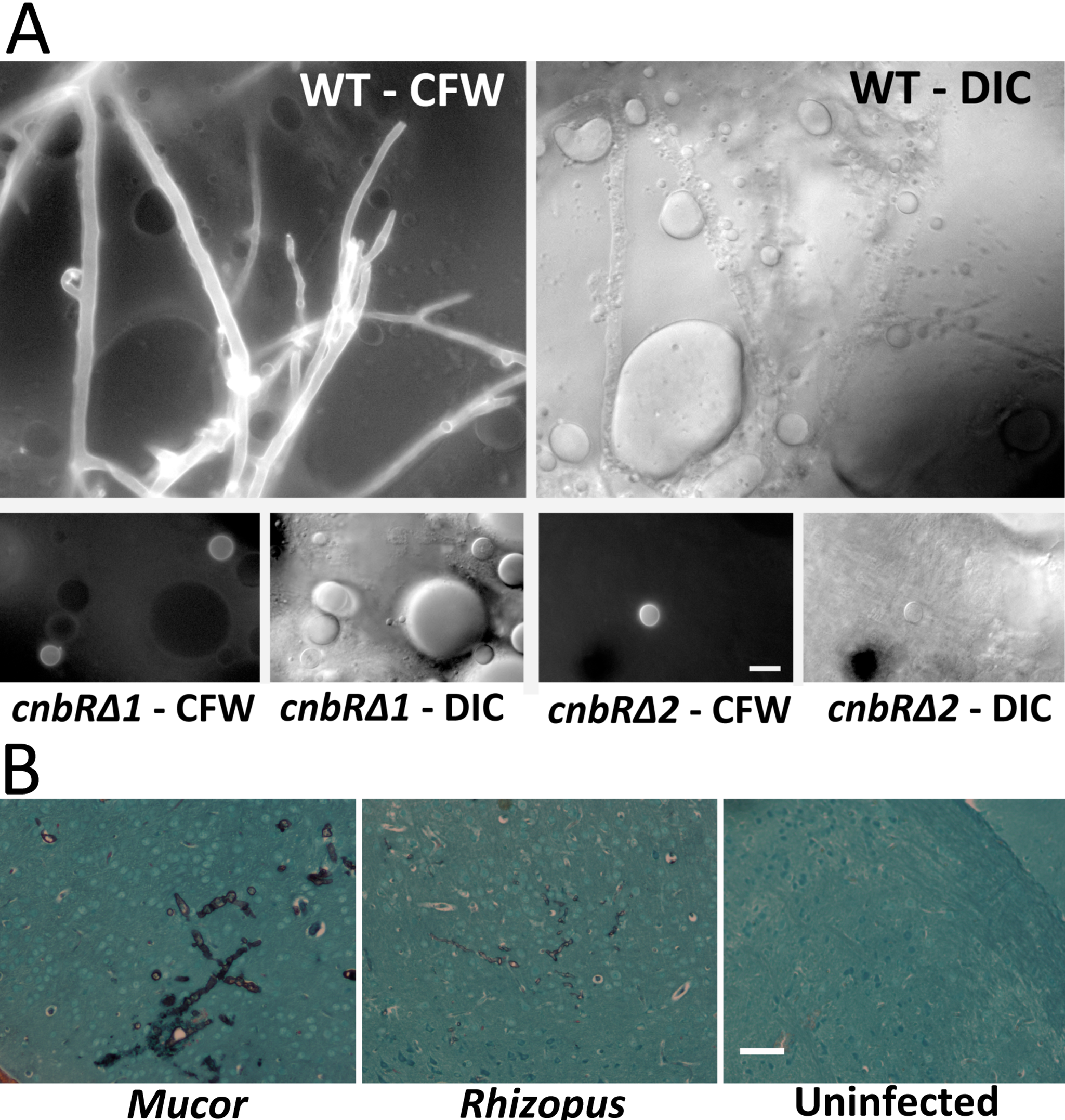

Supplement: Figure S10 — Morphology of M. circinelloides in infected host. (A) Wax moth larvae were infected with Mucor (20,000 spores or yeast) and after two days, the larvae were frozen and sliced for microscopic observation. Fungal mass in tissues was stained with a 0.05% solution of calcofluor white. The wild-type displayed hyphal growth inside of infected tissues, whereas the two independent cnbRΔ mutants only grew as yeast inside of the wax moth larva hosts. Scale = 20 µm. CFW: calcofluor white, DIC: differential interference contrast. (B) Mice were infected with Mucor or Rhizopus, and the brains from infected mice were collected and tissue specimens were stained with Gomori's methenamine silver (GSM) at 2 days post infection. Both fungi displayed hyphal growth in the recovered brain tissues. Scale = 50 µm. (TIF) [file ppat.1003625.s010.tif]

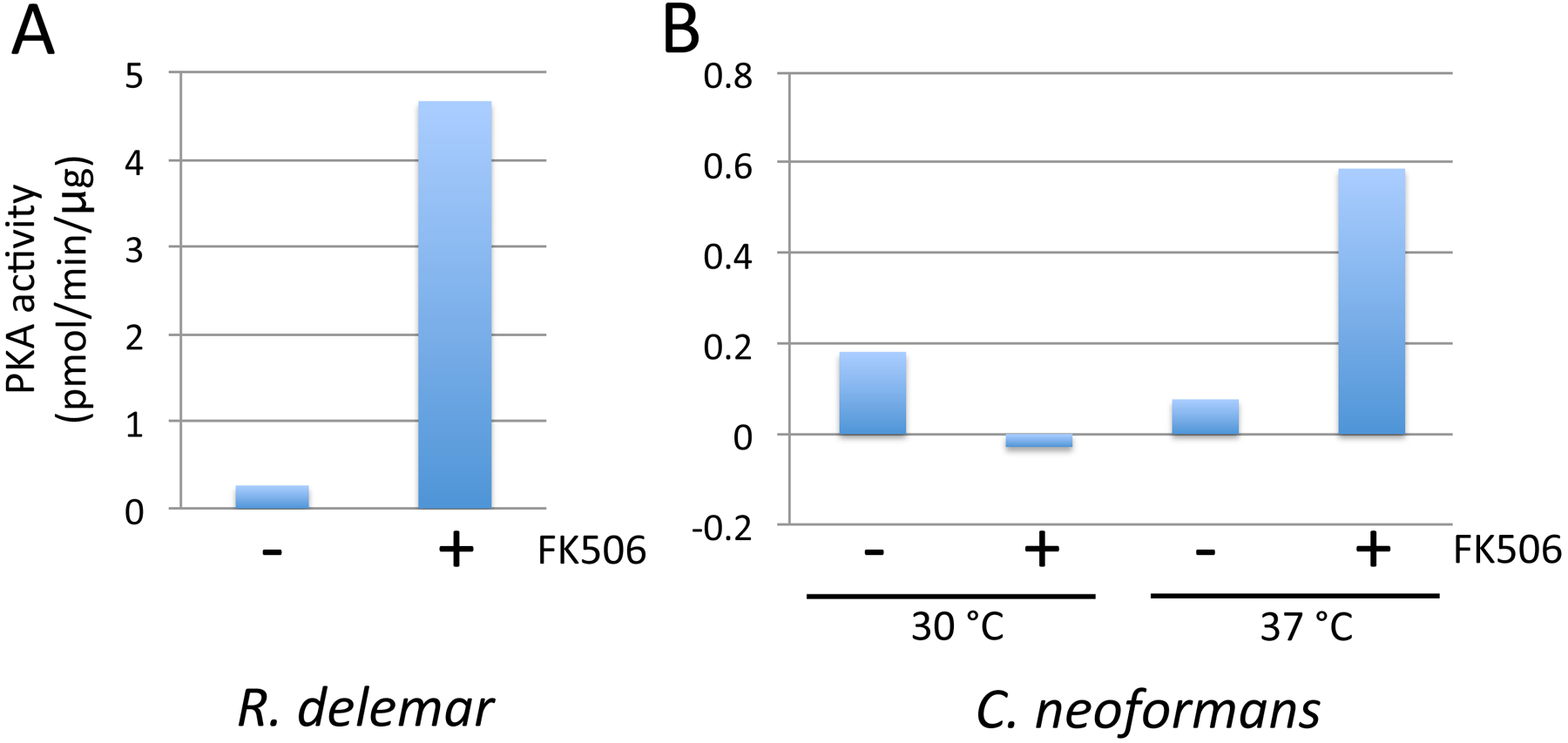

Supplement: Figure S11 — FK506 increased cAMP-dependent protein kinase A (PKA) activity in two other pathogenic fungi, R. delemar and C. neoformans. (A) When treated with FK506, PKA activity was elevated in R. delemar. (B) Calcineurin is known to function at 37°C in the basidiomycete pathogen C. neoformans. At this host temperature, FK506 treatment resulted in higher PKA activity. (TIF) [file ppat.1003625.s011.tif]

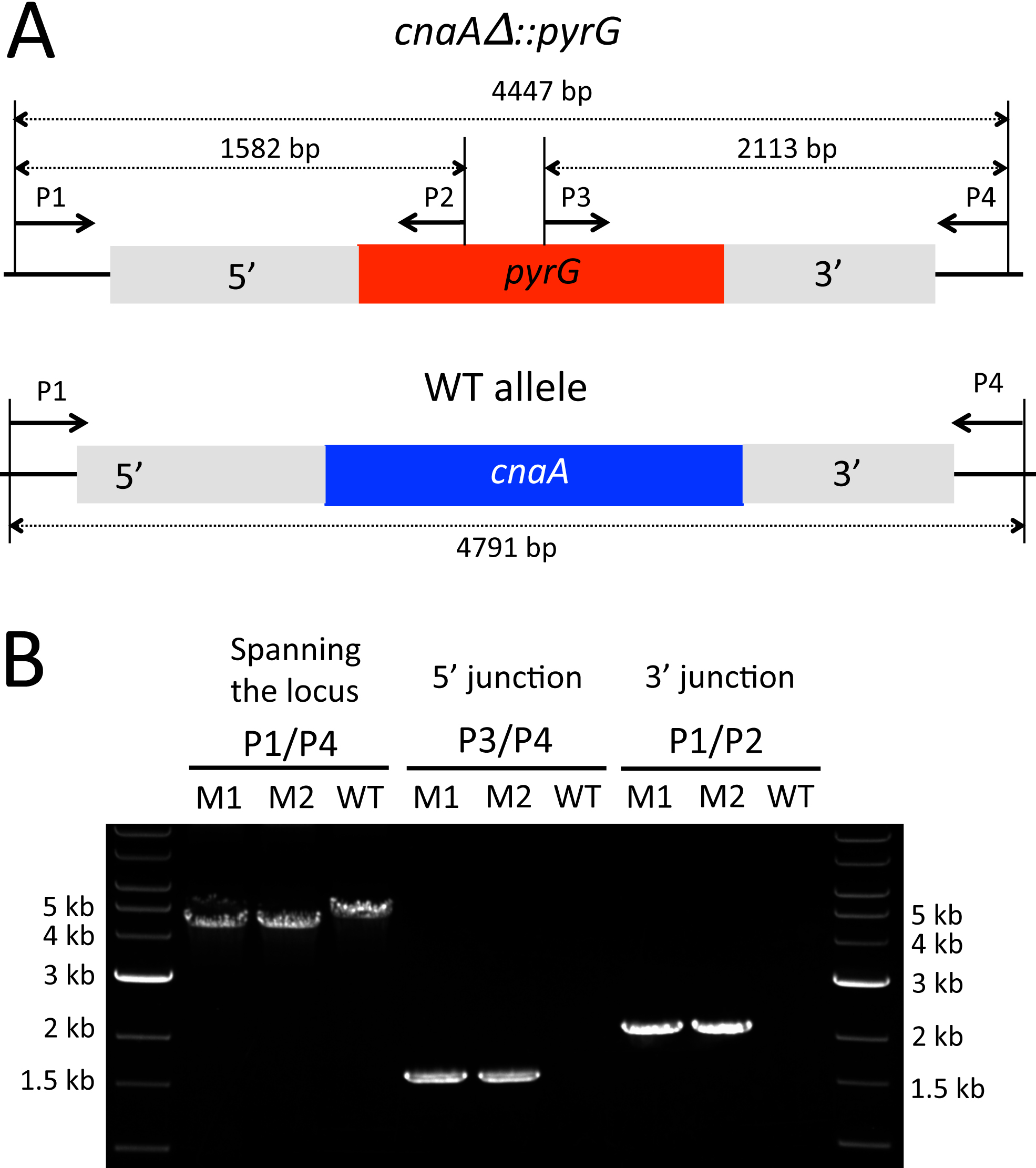

Supplement: Figure S12 — PCR confirmation of cnaA gene disruption. (A) Illustration of the cnaAΔ::pyrG and cnbA alleles with ∼1 kb of up- and downstream sequence. P1 and P4 primers recognize sequences outside of the disruption cassette. P2 and P3 primers recognize the pyrG gene. P1, JOHE26840; P2, JOHE37644; P3, JOHE37645; P4, JOHE26845 (Table S1). (B) P1 and P4 primers amplified a 4447 bp product for the cnaAΔ::pyrG allele from two independent cnaA mutants, whereas the same primers amplified only a 4791 bp cnaA fragment from wild-type. P1 and P2 primer amplified a 1582 bp region from the 5′ junction of the cnaAΔ::pyrG allele, and the P3 and P4 primers a 2113 bp product from the 3′ junction of the cnaAΔ::pyrG allele from the two cnaA mutants. The same pairs of primers did not produce junction fragments from wild-type. The gene and primer sizes are not to scale. M1: MSL9 and M2: MSL10 (Table 1). (TIF) [file ppat.1003625.s012.tif]

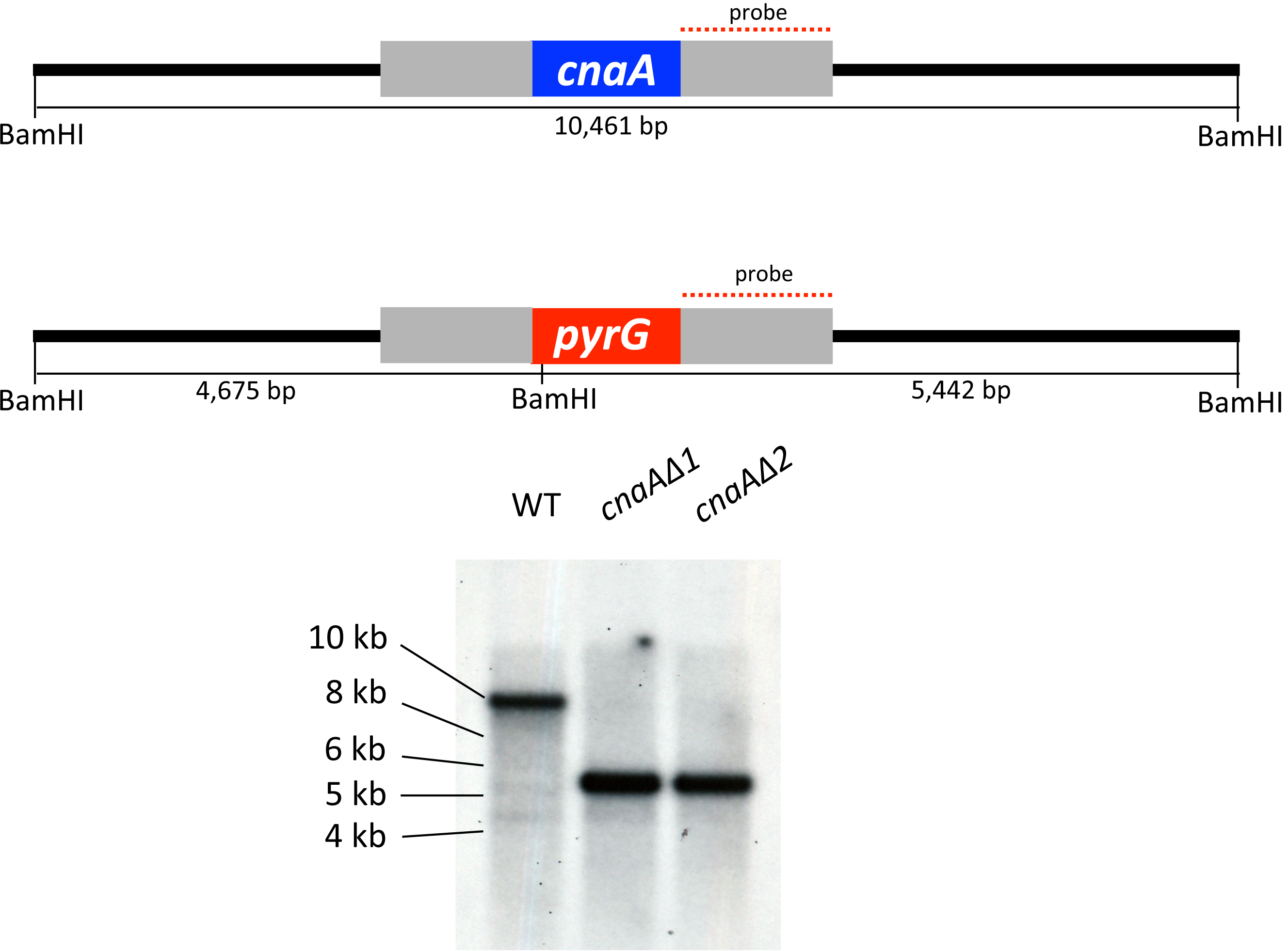

Supplement: Figure S13 — Southern blot confirmation of the disruption of the cnaA gene. Genomic DNA (30 µg) of wild-type (MU402), MSL9 (cnaAΔ1), and MSL10 (cnaAΔ2) were fully digested with BamHI. The 3′ UTR end of the cnaA gene was amplified and labeled with P32. The probe sequence is a part of the disruption cassette. The probe detected a 10,461 bp wild-type BamHI fragment, whereas a 5,442 bp BamHI fragment was detected in the cnaAΔ mutants, confirming a deletion of the cnaA gene in the cnaAΔ mutants. No apparent extra signals were detected, indicating that no ectopic integration events occurred. The gene sizes are not to scale. (TIF) [file ppat.1003625.s013.tif]

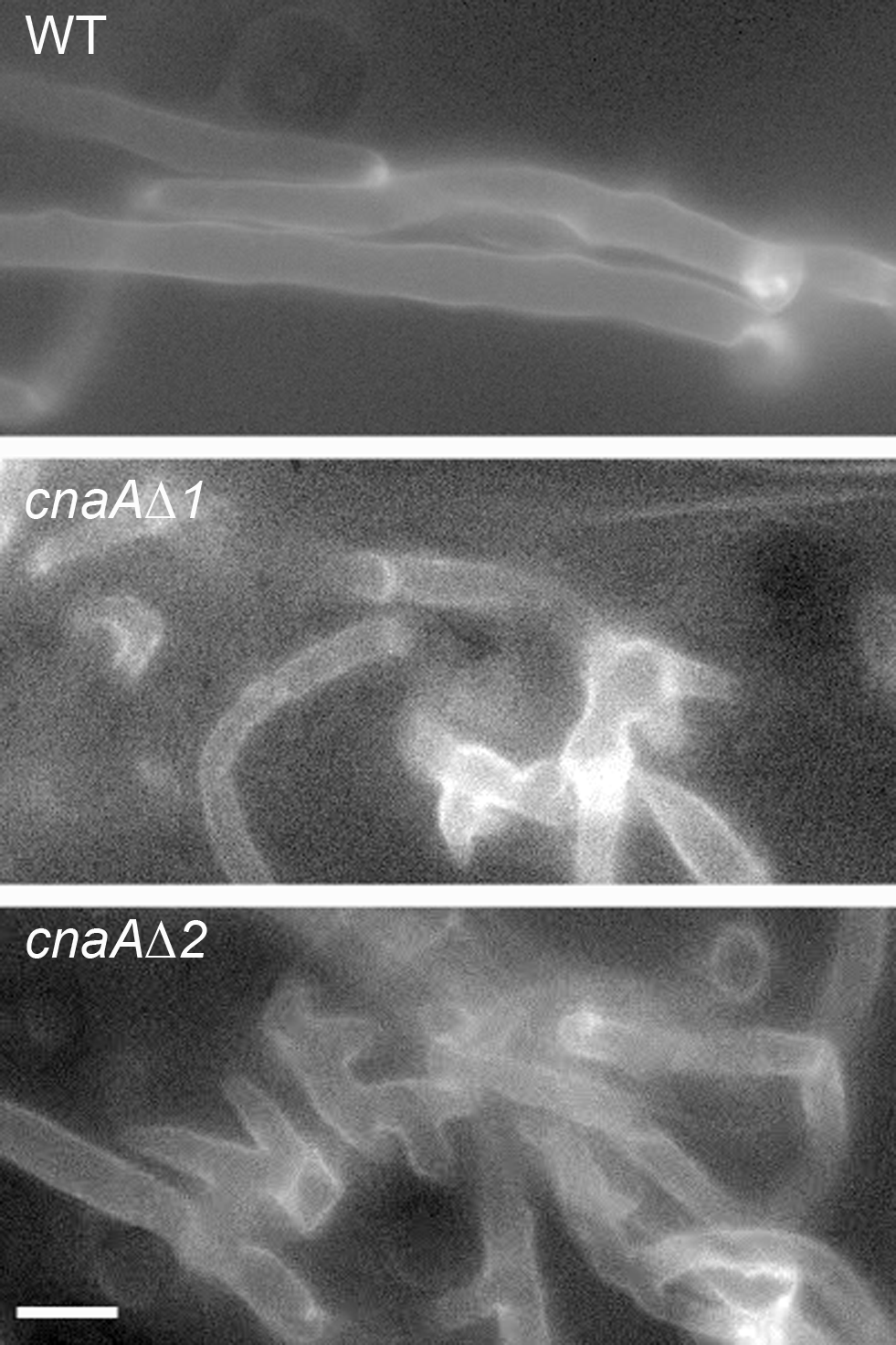

Supplement: Figure S14 — Morphology of cnaA mutants in the wax moth larva host. Wild-type and two independent cnaAΔ mutants (20,000 spores) were inoculated into wax moth larvae. After two days, the larvae were frozen and sliced for microscopic observation. A drop of 0.05% calcofluor white solution was applied to stain fungal mass in the tissues. Both of the wild-type and cnaAΔ mutants formed hyphae inside of the wax moth larva hosts. Scale = 10 µm. (TIF) [file ppat.1003625.s014.tif]

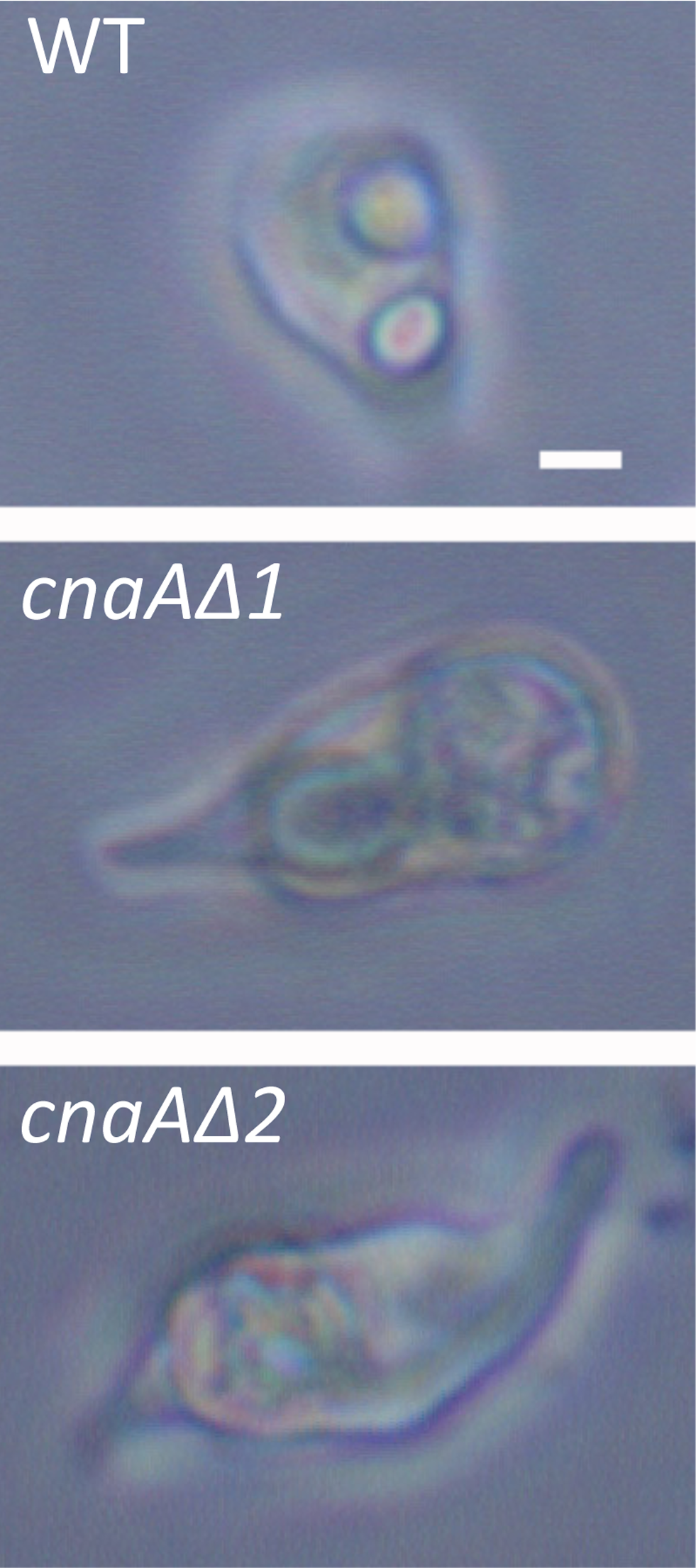

Supplement: Figure S15 — Larger spores of the cnaA mutants germinate earlier than wild-type. J774.A1 macrophage cells (5×10^5^) were inoculated into each well of a six-well plate. After 24 hours of incubation, 5×10^5^ spores were co-cultured with the macrophages. The interactions between macrophages and spores were observed every 30 minutes for 6 hours. The larger cnaA spores germinated earlier, at 3.5 hours, inside macrophages than the wild-type, at 6 hours. The images were taken at 3.5 hour. (TIF) [file ppat.1003625.s015.tif]

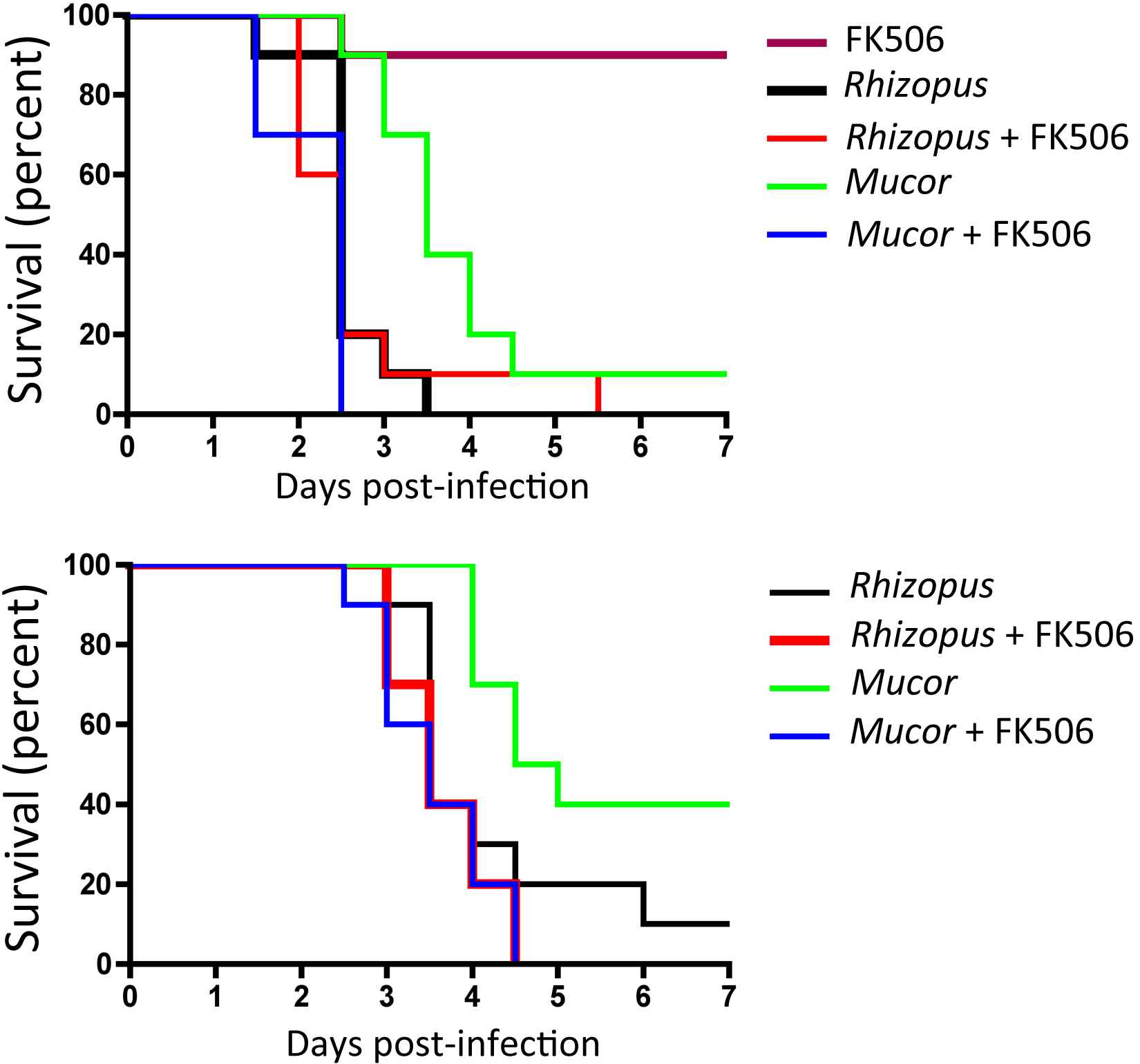

Supplement: Figure S16 — Effect of FK506 on diabetic (upper) and non-diabetic (bottom) murine hosts infected with M. circinelloides or R. oryzae. Groups of BALB/c mice were rendered diabetic with streptozocin (190 mg per body kg) through intraperitoneal injection 10 days prior to fungal challenge [53], [96]. The mice were infected with 104 Mucor (CNRMA04.805) spores or 103 Rhizopus (RA99-880) spores in 200 µL PBS via tail vein injection. The survival rate of the hosts was monitored twice a day, and body weight was measured daily. Animals that appeared moribund or in pain were sacrificed. The significance of mortality data was evaluated with Kaplan-Meier survival curves. The mice infected with Mucor or Rhizopus spores were immediately administered FK506 at a concentration of 5 mg per kg of body weight via intraperitoneal injection. Two additional FK506 treatments were performed at the same concentration at 24 and 48 hours post-infection. In both groups of mice infected with Mucor or Rhizopus, no apparent therapeutic effect of FK506 was observed. Non-diabetic murine host models were also tested, in which 105 Mucor spores and 104 Rhizopus spores were intravenously injected. FK506 at 5 mg per kg of body weight was administered at 0, 24, and 48 hours post-infection. No apparent therapeutic benefit of FK506 was observed in this analysis. A detrimental effect was observed in the mice infected with Mucor (p = 0.0015). It is possible that in both the diabetic and non-diabetic murine host models immunosuppression due to FK506 might have counteracted the antifungal effects of the drug. These results suggest that lower doses of FK506 (altered dosage regimens), less or non-immunosuppressive FK506 analogs, or a combination of FK506 with other antifungal drugs will be necessary for therapeutic efficacy. (TIF) [file ppat.1003625.s016.tif]

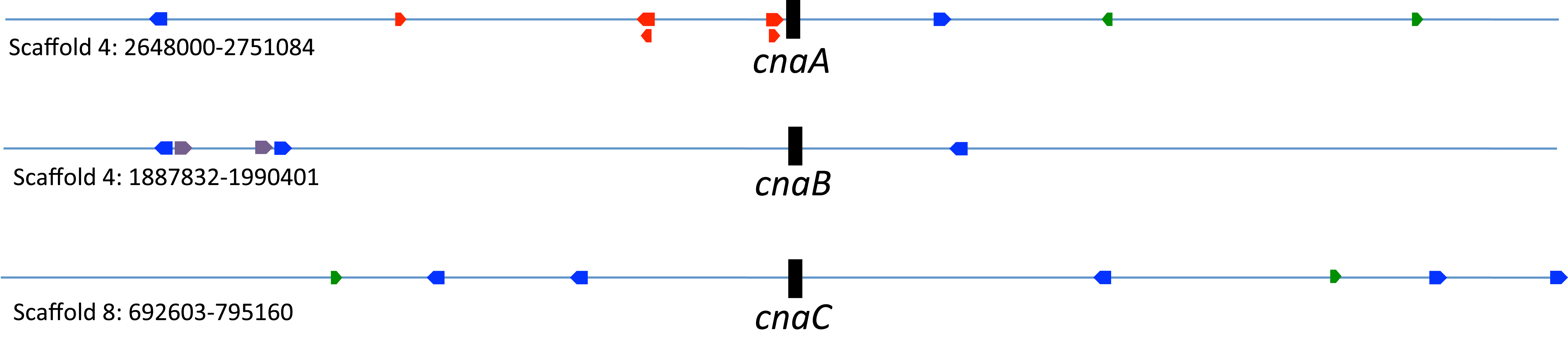

Supplement: Figure S17 — One hundred kilobases upstream and downstream of the three cna genes are depicted. The 5′ and 3′ flanking regions of the three cna genes are not syntenic; however, repetitive elements are present. The blue-arrow repetitive sequences (∼1 kb) are found in the flanking areas of all three cna genes; the green-arrow repetitive elements (∼500 bp) are found in the flanking areas of the cnaA and cnaC genes; the red-arrow and gray-arrow repetitive elements are found only in the flanking area of the cnaA or cnaB gene, respectively. These observations illustrate that the three cna genes may have evolved from multiple segmental gene duplication events, which may have been facilitated by these or other repetitive elements. (TIF) [file ppat.1003625.s017.tif]
