# Supplementary material for: Calcineurin Plays Key Roles in the Dimorphic Transition and Virulence of the Human Pathogenic Zygomycete Mucor circinelloides
Source: PLoS Pathog. 2013 Sep 5;9(9):e1003625. doi: 10.1371/journal.ppat.1003625 (PMC3764228; doi:10.1371/journal.ppat.1003625)
Supplement: Table S1 — Primers used in this study. (DOC) [file ppat.1003625.s018.doc]

**Table S1**. Primers used in this study

| Name | Sequences (5' to 3') | Remarks |
| --- | --- | --- |
| JOHE23748 | ATGGATGAGATTATTAGCCCAGCA | RT PCR forward primer to amplify *cnaA* |
| JOHE23750 | TCACTGAAACATGGCATGAACAACC | RT PCR reverse primer to amplify *cnaA* |
| JOHE23751 | ATGACAACATCTTCTAAATCACCAG | RT PCR forward primer to amplify *cnaB* |
| JOHE23753 | TTATATGTCGCTGACAATGAC | RT PCR reverse primer to amplify *cnaB* |
| JOHE23754 | ATGTCTGATATAGCCATTACAA | RT PCR forward primer to amplify *cnaB* |
| JOHE23756 | TTAGACTTGAGGTTGGGCTC | RT PCR reverse primer to amplify *cnaB* |
|  |  |  |
| JOHE20751 | TGGGTTGAAAGGGAAAAGAA | primer to sequence *cnaA* ORF |
| JOHE23765 | GTCCCAAGTAGCACCAAAGC | primer to sequence *cnaB* ORF |
| JOHE23766 | GCTGCTCCTTCTCATGCTCT | primer to sequence *cnaB* ORF |
| JOHE23767 | CACTTGGCTTGAAATCAGCA | primer to sequence *cnaC* ORF |
| JOHE23768 | ATGCTCCTGCATGTGTCAAA | primer to sequence *cnaC* ORF |
|  |  |  |
| JOHE22226 | TAAGGTCGATGTTGCCACTG | primer for *cnbR* disruption, 5’ region |
| JOHE22227 | CAAGTACCAATGCTGAGGCAAGTTGCGAACCAGATTGACC | primer for *cnbR* disruption, 5’ region |
| JOHE22228 | GGTCAATCTGGTTCGCAACTTGCCTCAGCATTGGTACTTG | primer for *cnbR* disruption, *pyrG* |
| JOHE22229 | GTGTGTGTGTATGGGGCAAAGTACACTGGCCATGCTATCG | primer for *cnbR* disruption, *pyrG* |
| JOHE22230 | CGATAGCATGGCCAGTGTACTTTGCCCCATACACACACAC | primer for *cnbR* disruption, 3’ region |
| JOHE22231 | CGAAAACAACAAACGCATTG | primer for *cnbR* disruption, 3’ region |
| JOHE22236 | CGGCAAGTACTGTGTCCTCA | nested primer for *cnbR* disruption |
| JOHE22237 | ATGGCAAAGTCGAAGAGGAA | nested primer for *cnbR* disruption, primer for probe for Southern blot |
| JOHE39531 | TTTGCCCCATACACACACAC | primer for probe for Southern blot |
| JOHE37644 | ACCCACTCACTTTCCATTCG | primer for *pyrG* to determine insertion |
| JOHE37645 | TGCTTTTGTTGGCTGAGATG | primer for *pyrG* to determine insertion |
|  |  |  |
| JOHE26840 | TTGGTCCCCAAAAAGTTGAG | primer for *cnaA* disruption, 5’ region |
| JOHE26841 | CAAGTACCAATGCTGAGGCATGGAACCAGAGAAACGAAAAA | primer for *cnaA* disruption, 5’ region |
| JOHE26842 | TTTTTCGTTTCTCTGGTTCCATGCCTCAGCATTGGTACTTG | primer for *cnaA* disruption, *pyrG* |
| JOHE26843 | CGAAACTGAATGACGAGGAAAGTACACTGGCCATGCTATCG | primer for *cnaA* disruption, *pyrG* |
| JOHE26844 | CGATAGCATGGCCAGTGTACTTTCCTCGTCATTCAGTTTCG | primer for *cnaA* disruption, 3’ region |
| JOHE26845 | GATTCATGGCATCCAGCTTT | primer for *cnaA* disruption, 3’ region |
| JOHE26846 | TGGTGGTCTAAAACGGCTTC | nested primer for *cnaA* disruption cassette |
| JOHE26847 | ACAGGTCATTCCGTCTCTGG | nested primer for *cnaA* disruption cassette, primer for probe for Southern blot |
| JOHE39530 | TTTCCTCGTCATTCAGTTTCG | primer for probe for Southern blot |
| JOHE24019 | AGAGGTTATTTCTCAATCGAGTGT | Real time PCR primer for *cnaA* |
| JOHE24020 | CTCTCGGTGTATTTATGACGAC | Real time PCR primer for *cnaA* |
| JOHE24021 | TTCCTGCGCCCATCACAGTA | Real time PCR primer for *cnaB* |
| JOHE24022 | CTCCAGAGATACAAAACGCACT | Real time PCR primer for *cnaB* |
| JOHE24023 | GGGGCTATTTCTCGATTGAATG | Real time PCR primer for *cnaC* |
| JOHE24024 | CTCATCAGAATACTTGGTTTGACA | Real time PCR primer for *cnaC* |
| JOHE24075 | ATGGTCGGTATGGGTCAAAA | Real time PCR primer for actin |
| JOHE24076 | GCCTCAGTCAAGAGGACAGG | Real time PCR primer for actin |
|  |  |  |
| JOHE20700 | CGCTTGTGGTGAGCTATTGA | primer for *cnbR* sequencing |
| JOHE20701 | GAGCGTGCCTGTCAGATGTA | primer for *cnbR* sequencing |
| JOHE22226 | TAAGGTCGATGTTGCCACTG | primer for *cnbR* sequencing |
| JOHE22231 | CGAAAACAACAAACGCATTG | primer for *cnbR* sequencing |
|  |  |  |
| JOHE20702 | TCAACTAACGCAGCTTACGG | primer for *cnaA* sequencing |
| JOHE20703 | CTGTGCTTCATGAGCTCGAA | primer for *cnaA* sequencing |
| JOHE20750 | ACGGTGGTCTTTCACCAGAG | primer for *cnaA* sequencing |
| JOHE20751 | TGGGTTGAAAGGGAAAAGAA | primer for *cnaA* sequencing |
| JOHE23458 | ATGAAGCATGCATGAACTCG | primer for *cnaA* sequencing |
| JOHE23459 | TCAAATCGTCCAATGTCTGC | primer for *cnaA* sequencing |
| JOHE23748 | ATGGATGAGATTATTAGCCCAGCA | primer for *cnaA* sequencing |
| JOHE23764 | CCCTTCATTTCAAGTCCGTTT | primer for *cnaA* sequencing |
| JOHE24082 | CGAAACAAAATCATGGCTGTT | primer for *cnaA* sequencing |
| JOHE26845 | GATTCATGGCATCCAGCTTT | primer for *cnaA* sequencing |
|  |  |  |
| JOHE20704 | GTTTGGCGAGGAAACAACAT | primer for *cnaB* sequencing |
| JOHE20705 | CATGATCAGCGTTCTGTGCT | primer for *cnaB* sequencing |
| JOHE23460 | TGTAGGCAGACACAAGTATTCTGA | primer for *cnaB* sequencing |
| JOHE23751 | ATGACAACATCTTCTAAATCACCAG | primer for *cnaB* sequencing |
| JOHE23753 | TTATATGTCGCTGACAATGAC | primer for *cnaB* sequencing |
| JOHE23766 | GCTGCTCCTTCTCATGCTCT | primer for *cnaB* sequencing |
| JOHE24011 | GCATTACCATTGGGTGCTTT | primer for *cnaB* sequencing |
| JOHE24012 | GGTCGTCCTCTGCAAGTAGC | primer for *cnaB* sequencing |
| JOHE26869 | CCAAGACGCTCTTTTCAAGC | primer for *cnaB* sequencing |
|  |  |  |
| JOHE20706 | GCACAAGCCAATGGGTAAGT | primer for *cnaC* sequencing |
| JOHE20707 | TCGCCAGACAAGTTCTTGAG | primer for *cnaC* sequencing |
| JOHE23462 | GCTTTGACAGCCTTCCTTTG | primer for *cnaC* sequencing |
| JOHE23463 | TGCCTCTTCAATGTCTTTGAGT | primer for *cnaC* sequencing |
| JOHE23754 | ATGTCTGATATAGCCATTACAA | primer for *cnaC* sequencing |
| JOHE23755 | ATGGAGGATGGCAGTGTCGT | primer for *cnaC* sequencing |
| JOHE23756 | TTAGACTTGAGGTTGGGCTC | primer for *cnaC* sequencing |
| JOHE23767 | CACTTGGCTTGAAATCAGCA | primer for *cnaC* sequencing |
| JOHE23768 | ATGCTCCTGCATGTGTCAAA | primer for *cnaC* sequencing |
| JOHE24467 | GACCTGAAGCGATCAGGATG | primer for *cnaC* sequencing |
| JOHE24468 | GGAACAGTGGCATCAGACAA | primer for *cnaC* sequencing |
